# The Effect of Flake Production and *In Vitro* Digestion on Releasing Minerals and Trace Elements from Wheat Flakes: The Extended Study of Dietary Intakes for Individual Life Stage Groups

**DOI:** 10.3390/nu15112509

**Published:** 2023-05-28

**Authors:** Daniela Sumczynski, Miroslav Fišera, Richardos Nikolaos Salek, Jana Orsavová

**Affiliations:** 1 Department of Food Analysis and Chemistry, Tomas Bata University in Zlín, Vavrečkova 5669, 760 01 Zlín, Czech Republic; 2 Department of Food Technology, Tomas Bata University in Zlín, Vavrečkova 5669, 760 01 Zlín, Czech Republic; 3 Language Centre, Tomas Bata University in Zlín, Štefánikova 5670, 760 01 Zlín, Czech Republic

**Keywords:** mineral, essential trace element, toxic trace element, *in vitro* digestion, dietary intake evaluation, life stage group, metal pollution index, wheat flake, retention factor, disease prevention

## Abstract

This thorough study analyses the amounts of 43 minerals and trace elements in non-traditional wheat grains, flakes, and undigested flake portions using ICP-MS and establishes declines in their respective contents after the flake production. It also identifies appropriate dietary intakes, *in vitro* digestibility values, retention factors, and metal pollution indexes. The element contents in wheat flakes are lower than in wheat grains after the hydrothermal treatment process, and their declines are: Na (48–72%), Ce (47–72%), Sr (43–55%), Tl (33–43%), Ti (32–41%), U (31–44%), Ho (29–69%), Cr (26–64%), Zr (26–58%), Ag (25–52%), and Ca (25–46%). The flakes significantly contributed to the recommended dietary intake or adequate intake of particular elements for men of all categories as follows: Mn (143%) > Mo > Cu > Mg ≥ Cr > Fe (16%); for women: Mn (up to 183%) > Mo > Cu > Cr ≥ Mg > Fe (7–16%); for pregnant women aged 19–30: Mn (165%) > Mo > Cu > Mg > Cr (25%); and finally, for lactating women: Mn (127%) > Mo > Cu > Mg > Cr (17%). The contributions to the provisional tolerable weekly or monthly intakes of all toxic elements were established as being within the official limits. The daily intakes for non-essential elements were also calculated. The retention factors were calculated to assess the element concentrations in the undigested part using the digestibility values (87.4–90.5%). The highest retention factors were obtained for V (63–92%), Y (57–96%), Ce (43–76%), Pb (34–58%), Tl (32–70%), Ta (31–66%), and Ge (30–49%). K, Mg, P, Zn, Ba, Bi, Ga, Sb, Cu, Ni, and As appear to be released easily from flake matrices during digestion. The metal pollution index has been confirmed as being lower for non-traditional wheat flakes when compared with grains. Importantly, 15–25% of the metal pollution index assessed for native flakes remains in the undigested flake portion after *in vitro* digestion.

## 1. Introduction

Whole-grain cereal flakes are one of the most popular sources of value-added ingredients, such as vitamins, minerals, dietary fibre, phenolics, and proteins. Since cereal flakes are commonly consumed around the world, they contribute to enhanced health attributes, including a controlled blood glucose level and a reduced risk of obesity and cardiovascular diseases [1,2,3].

Common minerals and trace elements in whole-grain foods include P, Mn, Mo, Cu, Fe, Zn, and Ni. They are mostly covalently bound to the cell wall in the grain outer layers. Processing technologies, such as milling and hydrothermal treatment technology, have been applied to release elements from these outer layers. Unfortunately, hydrothermal treatment may reduce the amount of various bioactive substances depending on the grain type, grain matrix, and other ambient factors [1,2,3,4,5,6]. Whole grains contain more minerals and trace elements than refined grains or flakes [7]. Importantly, a high consumption of cereal grains may cause a deficit in the absorption of elements, such as Zn, Ca, Mg, Mn, K, Cu, and Fe, due to the presence of element antinutrients, including phytic acid, phenolics, and tannins. As a consequence, minerals chelated with phytic acid are biologically unavailable, ultimately leading to an impaired micronutrient status, growth, and development and even increased mortality [7,8,9,10]. The intake of flakes rich in minerals is not necessarily correlated with large amounts of minerals being absorbed in the digestive tract, as possible interactions between the components of the food matrix limit their complete release. During digestion, the release of substances from the food matrix is influenced by various factors, including biochemical (presence of bile salts, enzymes, and antinutrients) and physicochemical (acidic or basic pH, temperature, and chemical form of the bound element) factors [1,11]. 

Therefore, since the element content after the digestion may significantly differ from its original amount in the source, it is important to quantify the available proportion of digestible and indigestible elements. Bioaccessibility and bioavailability are the main factors for evaluating the efficiency of the element intake from the food matrix and its further nutritional value for the organism [10,12]. The first one, bioaccessibility, expresses the quantity of digested substances released during the digestion. It also determines their stability throughout the process and monitors their concentrations released into the targeted cell to show their biological activity [1,6,8,10,11,12]. The bioavailability of minerals and trace elements can be then defined as the proportion of ingested minerals absorbed and available for metabolic functions. Bioavailability is a technical term explaining that not 100% of the ingested nutrients are absorbed. Considering minerals, it varies from less than 1% to more than 90% [8] and correlates with their bioaccessibility. Throughout time, *in vitro* digestion models have been gaining importance and are employed to monitor changes in the element profiles during the digestion processes and to predict bioaccessibility [6,11]. Furthermore, an innovative study has analysed solid undigested proportions of food of plant origin to calculate the remaining amounts of bioactive compounds after *in vitro* digestion simulation [13]. This remaining factor represents the percentage of analytes that are retained in the undigested matrix.

To the best of our knowledge, only a few studies have determined the differences in the concentrations of minerals and trace elements in the production of non-traditional wheat flakes and also in their residual concentrations after *in vitro* digestion with respect to the dietary reference intakes defined for essential, non-essential, and toxic elements. Data discussing the contribution of non-traditional wheat flakes to the RDA (recommended dietary allowances), AI (adequate intake), PTWI (provisional tolerable weekly intake), and PTMI (provisional tolerable monthly intake) for individual life stage groups has been very scarce, especially in the context of determining the contents of elements remaining in the undigested flakes. Additionally, the MPI (metal pollution index) has recently become one of the main studied aspects as it expresses the level of risk for human health. That is why this experiment provides a thorough analysis, examining all factors essential to evaluate the quality and safety of non-traditional wheat flakes for adults, seniors, and pregnant and lactating women of a specified body weight.

## 2. Materials and Methods

### 2.1. Reagents

Pepsin (E.C. 3.4.23.1), with an activity of 0.7 FIG-U/mg, and an enzyme mixture of pancreatin with activities of 350 FIG-U/g for protease, 7500 FIG-U/g for amylase, and 6000 FIG-U/g for lipase were purchased from Merck (Darmstadt, Germany). ICP-MS 25-standards (Li, Be, B, Mg, Al, Sc, Mn, Co, Ni, Cu, Zn, Ga, Sr, Y, Mo, Ag, Cs, Ba, Ce, Tb, Ho, Ta, Tl, Bi, and U), ICP-MS 18-standards (Na, P, S, K, Ca, Ti, V, Cr, Fe, Ge, As, Se, ^90^Zr, Cd, Sn, Sb, Hg, and Pb), and ICP-MS internal In and Rh standards were acquired from Analytika (Prague, Czech Republic). Argon and helium were obtained from Linde Gas (Zlín, Czech Republic), and ultrapure water was supplied by a Purelab Classic Elga water system (Labwater, London, UK). Certified reference materials (CRMs) of Metranal^®^8 and NIST rice flour 1568b were purchased from Analytika Ltd. (Prague, Czech Republic); CRM lichen was bought via the International Atomic Energy Agency (Vienna, Austria). Tune 7 and 8 solutions were purchased from Analytika (Prague, Czech Republic).

### 2.2. Sample Collection

Five non-traditional wheat grain samples collected from Bäckerhaus Veit, GmbH in Bempflingen (Germany) were harvested in the Baden-Wittenberg agricultural area in 2021. They were labelled as Samtrot and Tirol (both *Triticum compactum* L.), Dickkopf (*Triticum aestivum* × *Triticum spelta* Schlegel cross), Rotkorn (red wheat *Triticum aestivum* var. *milturum*), and Megali (*Triticosecale* spp.).

### 2.3. Production of Non-Traditional Wheat Flakes

Non-traditional wheat grains (500 g) were cooked in 2.5 L hot water (ultrapure water, 18.2 MΩ.cm, Purelab Classic Elga system, LabWater, London, UK) at a stable temperature of 95 °C. Megali grains were cooked for 20 min, Samtrot, Tirol, and Dickkopf grains for 15 min, and Rotkorn red grains for 10 min. The cooking time corresponded to the condition in which the grain is suitable for consumption. After being conditioned at room temperature for 5–10 min, the grains were rolled using a Combi-Star mill grinder (Waldner Biotech, Lienz, Austria) to obtain flakes with a thickness of 0.65–0.75 mm [14]. Subsequently, the flakes were dried in a laboratory oven (Venticell, BMT Medical Technology, Brno, Czech Republic) at 80 °C for 2 h until the dry matter reached 90% [15]. Prior to the analysis by ICP-MS, both non-traditional grains and flakes were stored in dark plastic bottles at 20 °C, with the storage period limited to 2 months.

### 2.4. Simulated In Vitro Gastrointestinal Digestion

The *in vitro* digestion process, including gastric and intestinal digestion, was performed according to [13] with a slight modification. Dry matter and ash content were determined following [15]. The *in vitro* digestibility of native wheat flake parts was initially assessed using pepsin and later by a mixture of pancreatin enzymes in a Daisy incubator (Ankom Technology, Macedon, NY, USA). First, the samples were weighed (0.25 g) and sealed (impulse sealer KF-200H, Penta Servis, Holice, Czech Republic) into F57 bags (Ankom Technology, Macedon, NY, USA). To simulate gastric processes, the polyethylene incubation bottle was filled with 1.7 L of 0.1 M HCl containing pepsin (3.0 g). The samples were incubated for 2 h at 37 °C and then rinsed with ultrapure water. For intestinal conditions, pH 7.45 phosphate buffer (32.50 g of Na_2_HPO_4_.12 H_2_O and 3.09 g of KH_2_PO_4_ dissolved in 1.7 L of ultrapure water) and a mixture of pancreatin enzymes (3.0 g) were added to the incubation bottle. After being incubated at 37 °C for 24 h, the samples were placed into an oven (Venticell, BMT Medical Technology, Brno, Czech Republic) at 80 °C for 30 min to release gelatinization starch. After the incubation, the samples were rinsed multiple times with ultrapure water, dried at 105 °C for 24 h, and weighed. Finally, the samples were combusted in a muffle furnace (LM112.10, Veb Elektro, Berlin, Germany) at 550 °C for 5.5 h, cooled, and weighed. *In vitro* digestibility, expressed as the DMD value (dry matter digestibility), was calculated using Equations (1)–(5):

(1) $$DMD(\%)=100-\frac{100\times DMR}{m2\times DM}$$

(2) DMR=m3−m1×c1

(3) $$DM=\frac{DW\times ms}{100}$$

(4) $$c1=\frac{ms}{m1}$$

(5) $$c2=\frac{mp}{m1}$$

where DMD is the dry matter digestibility (%), DMR is the sample weight without the sack after the digestion and drying (g), DM is the sample dry weight (g), DW is the sample dry weight (expressed in %), ms is the sample weight for dry matter determination (g), c1 is the correction of the weight of the sack after the incubation (g), c2 is the correction of the weight of the sack after the combustion (g), mp is the weight of ash from the empty correction sack (g), m1 is the weight of the empty bag (g), m2 is the sample weight (g), m3 is the weight of the dried bag with the sample after the incubation (g).

### 2.5. Preparation of Undigested Parts of Non-Traditional Wheat Flakes

To obtain undigested residues of the non-traditional wheat flakes, the digestibility assessment process was terminated by drying the samples at 105 °C for 24 h. Solid undigested residues of the flakes were decomposed using 67% Analpure® HNO_3_.

### 2.6. ICP-MS Analysis 

#### 2.6.1. Sample Preparation

Forty-three minerals and trace elements were identified in raw wheat grains and in the native and undigested parts of non-traditional wheat flakes. Ultrapure water of 18.2 MΩcm was obtained from Purelab Classic Elga equipment (LabWater, London, UK). Seven millilitres of 67% Analpure® HNO_3_ and 1 mL of 30% Analpure® H_2_O_2_ were placed onto a 0.20 g sample and decomposed into polytetrafluoroethylene (PTFE) vessels using a microwave system (Milestone Ethos One, Sorisole, Italy), with the parameters set as follows: 500 W for 10 min at 150 °C, 1500 W for 15 min at 180 °C, and finally 500 W for 15 min at 150 °C. After being cooled, the final sample volume was adjusted to 25 mL using ultrapure water. The final samples were promptly analysed using ICP-MS [16].

#### 2.6.2. Quality Control

The daily performance of ICP-MS in terms of sensitivity and background signals were tested using Tune 7 and 8 solutions with Ag, Al, Ba, Be, Bi, Ce, Co, Cs, Cu, Ga, Ho, In, Li, Mg, Mn, Ni, Rh, Sc, Sr, Ta, Tb, Tl, U, Y, and Zn containing 1 μg/L of each element in 2% HNO_3_ and Ba, Bi, Ce, Co, In, Li, and U containing 1 μg/L of each element in 2% HNO_3_ + 0.5% HCl. To obtain calibration curves, two sets of internal standards were prepared to match the concentration ranges in the samples: a high standard series of 25 elements (^7^Li, ^9^Be, ^11^B, ^24^Mg, ^27^Al, ^45^Sc, ^55^Mn, ^59^Co, ^60^Ni, ^63^Cu, ^66^Zn, ^71^Ga, ^88^Sr, ^89^Y, ^95^Mo, ^107^Ag, ^133^Cs, ^137^Ba, ^140^Ce, ^159^Tb, ^165^Ho, ^181^Ta, ^205^Tl, ^209^Bi, and ^238^U) at concentrations of 3–35 µg/L and a low standard series of 18 elements (^23^Na, ^31^P, ^32^S, ^39^K, ^44^Ca, ^48^Ti, ^51^V, ^52^Cr, ^57^Fe, ^73^Ge, ^75^As, ^77^Se, ^90^Zr, ^111^Cd, ^118^Sn, ^121^Sb, ^202^Hg, and ^208^Pb) with concentrations between 0.5 and 1.0 µg/L. As internal standards, rhodium (^103^Rh) and indium (^115^In) were used at concentrations of 10 and 5 µg/L, respectively. Certified reference materials (CRM) of green algae Metranal^®^8 and NIST Rice flour 1568b from the National Institute of Standards and Technology (both purchased via Analytica Ltd., Prague, Czech Republic) and lichens supported by the Atomic Energy Agency (Vienna, Austria) were applied to evaluate the measurement accuracy (Table S1).

#### 2.6.3. ICP-MS Instrumentation

Analyses were performed using a Thermo Scientific iCAP Qc inductively coupled plasma mass spectrometer (Thermo Scientific, Waltham, MA, USA). Furthermore, a collision cell (QCell) containing helium was applied to remove undesirable molecule ions by distinguishing their kinetic energy (CCT, collision cell technology; KED, kinetic energy discrimination mode). Specific working parameters were established as follows: power of 1550 W, sampling depth of 5 mm, cool gas flow rate of 14.0 L/min, auxiliary gas flow rate of 0.8 L/min, nebulizer gas flow rate of 1.015 L/min, helium flow rate of 4.1 mL/min, nebulizer pump speed of 40.00 rpm, and chamber temperature of 2.7 °C. The samples were analysed five times [16].

### 2.7. Effect of the Thermal Treatment and Flaking Process on the Mineral and Trace Element Content of Wheat Flakes

The decrease value (DV) in the concentrations of individual elements after the production of non-traditional wheat flakes was calculated according to the following equation and expressed as a percentage (%):

(6) $$DV=100-\frac{CF\times 100}{CRG}$$

where DV is the decrease in the analyte concentration within the flake production (%), CF is the analyte concentration in the flakes (ng or µg/g), and CRG is the analyte concentration in the raw grain (ng or µg/g).

### 2.8. Contribution of Elements to the RDA, AI, PTWI, and PTMI Values 

Daily levels of dietary intakes for minerals and trace elements from non-traditional wheat flakes were established and compared with the appropriate RDA values (recommended daily allowance) or AI* (adequate intake) as recommended by the Institute of Medicine [17,18,19,20]. The intake levels of toxic elements were also evaluated and compared with the PTWI (provisional tolerable weekly intake) or PTMI values (provisional tolerable monthly intake) as suggested by the FAO/WHO [21,22,23,24]. Appropriate levels of dietary intakes were determined for young adults (aged 19–30), middle-aged adults (aged 31–50), old-aged adults (aged 51–70), seniors (aged over 70), and pregnant and lactating women (aged between 19 and 30). When evaluating the dietary intake of toxic elements, the women’s and men’s body weight (bw) averages of 60, 70, 80, and 90 kg were included in this study.

The prescribed amounts for the RDA, AI*, PTWI, and PTMI* values for individual minerals and trace elements are specified in Table S2. Since there is no recommendation for the daily intake of non-traditional wheat flakes, the daily serving size was set to 100 g for each examined life stage group. 

### 2.9. Effect of In Vitro Digestion on the Element Content of Wheat Flakes

The amounts of all analytes that remained in the undigested portion of the wheat flakes were evaluated as the retention factors (RF), and the results were expressed as a percentage (%). The RF value was calculated using Equation (7):

(7) $$RF=\frac{CUWF\times (100-DMD)}{CNWF}$$

where RF is the retention factor of the appropriate analyte in the undigested part of the sample (%), CUWF is the analyte concentration in the undigested part of the wheat flakes (ng or µg/g), DMD is the dry matter digestibility value of the flakes (%), and CNWF is the analyte concentration in the native form of the flakes (ng or µg/g) [13].

### 2.10. Metal Pollution Index Evaluation 

To examine the total heavy metal concentrations in wheat grains, flakes, and undigested parts of the flakes, the metal pollution index (MPI) was calculated. This index was calculated using the geometric mean of the concentrations of all metals contained in the flakes [25,26]:

(8) $$MPI={\left(C1\times C2\times \ldots .Cn\right)}^{1/2}$$

where MPI is the metal pollution index (-), and C1, C2 …and Cn are the concentrations of an individual metal in the native grains, flakes, and undigested parts of the flakes.

To evaluate which part of the MPI value is maintained in the undigested part compared with native flakes, the remaining parts of the metal pollution index (RP_MPI_, %) were calculated as follows:

(9) $${RP}_{MPI}=\frac{{MPI}_{UWF}\times (100-DMD)}{{MPI}_{NF}}$$

where RP_MPI_ is the remaining part of the MPI index maintained in the undigested part of the flakes (%), MPI_UWF_ is the metal pollution index of the undigested part of the flakes (-), DMD is the dry matter digestibility of the flakes (%), and MPI_NF_ is the metal pollution index in the native flakes (-).

### 2.11. Statistical Analysis

Mineral and trace analyses were performed 5 times; the digestibility assay and the dry matter and ash content analyses were repeated 3 times. The results were expressed as mean ± standard deviation on a dry weight basis and were statistically evaluated using one-way analysis of variance (ANOVA, TriloByte Statistical software, Pardubice, Czech Republic). Subsequently, Tukey’s test was used to identify differences between the mean values; the level of significance was set to 5% (*p* < 0.05).

## 3. Results and Discussion

### 3.1. Dry Matter and Ash Contents and Digestibility Assessment of Non-Traditional Wheat Flakes 

To establish the dry matter digestibility (DMD), the dry matter and ash contents of the wheat flakes were assessed. As can be seen in Table S3, the dry matter varied between 91.6 and 93.8%. All analysed samples met the requirements in [27] for obtaining a moisture content in the flakes of less than 14%. The ash content was recorded as being between 1.82 and 1.94%. According to our previous study, the ash content in wheat flakes ranged from 1.7 to 2.4%, with the highest amounts being measured in kamut and spelt flakes [28]. *In vitro* digestibility was established using the enzymatic–gravimetric method, employing pepsin and pancreatin enzymes. Considering the flake production process, the DMD values ranged from 87.4 to 90.5%. Steam flaking, a process for cereal grains involving heat, moisture, and pressure application, may effectively improve the nutritional value of grains by increasing starch and protein digestibility [29].

### 3.2. Mineral and Trace Element Content of Non-Traditional Wheat Grains

Forty-three elements were identified using ICP-MS in non-traditional wheat grains of Samtrot, Tirol, Dickkopf, Rotkorn, and Megali varieties, their flakes, and undigested parts. Table 1 and Table 2 summarise their amounts (µg or ng/g) of dry weight. In the last few decades, numerous research papers have monitored the content of not only essential elements but also toxic elements of cereal grains [25,30,31,32]. It is generally known that the mineral and element content in cereals is affected by the cultivar, fertilization, soil, and local environmental conditions or by grain treatments, including steaming and flaking. As can be seen in Table 1, elements were established in the following order based on their maximum concentration values in grains: Mg (up to 1090 µg/g) > K > Ca > P > Mn > Fe > Zn > Cu > S > Na > Ti > Al > Sr (up to 2.76 µg/g) > Ba (up to 1140 ng/g) > Mo > Sn > Ni > B > Cr ≥ V > Pb ≥ Sc > Se > Li > Cd > Zr > Ce ≥ Co > Hg > As ≥ Ga > Ta > Ag > Sb ≥ Ge > Bi > Cs > Y ≥ U > Tb > Ho > Be > Tl (up to 0.09 ng/g). Next, toxic elements have been monitored in cereals for many years. A regular Cd intake supports such an accumulation of it and increases the risk of kidney disease, osteoporosis, and cancer [33]. Even though the average Cd content in cereals consumed in the EU varies only from 0.4 to 6.0 µg/100 g, the maximum Cd value in cereals has been set to 100 µg/kg [34,35]. More recently, the Scientific Panel on Contaminants in the Food Chain (the CONTAM Panel) has established maximum Cd contents of 100 µg/kg for cereal grains excluding wheat and rice and 200 µg/kg for wheat and rice grains and wheat bran designated for a direct consumption [36]. As the results show, the Cd concentrations were established as being in lower amounts than set by this limit. Commission Regulation (EU) No 420/2011 [37] and Commission Regulation (EU) 2021/1317 [38] define the maximum Pb level in cereals and legumes to 200 µg/kg, since Pb is harmful to the nervous system and causes blood disorders. Similarly, none of our samples exceeded this limit. The maximum concentrations of the remaining toxic elements, including Sn, Hg, and Ni, have not been established yet, except for an inorganic form of As, which is allowed up to a maximum limit of 200 µg/kg in non-parboiled milled rice (both polished and white rice) [39]. 

### 3.3. Mineral and Trace Element Contents of Non-Traditional Wheat Flakes

Cereal flakes constitute a wide range of products including ready-to-eat breakfast cereals suitable for a direct consumption or hot breakfast cereals requiring heat processing [3,40]. The mineral and trace element contents in non-traditional wheat flakes are presented in µg or ng per gram of dry weight (Table 1). This research has shown that the mineral and trace element content of wheat flakes is lower than in grains and is determined as being in the following order based on the maximum concentration values: Mg (up to 967 µg/g) > K > Ca > P > Mn > Fe > Zn > S ≥ Cu > Na > Ti ≥ Al (up to 2.01 µg/g) and Sr (up to 1430 ng/g) > Ba > Mo > Sn > B > Ni > V > Cr > Sc > Pb > Se > Cd > Li > Zr > Co > Ga ≥ Hg > As > Ta > Ce > Sb > Ag ≥ Ge > Bi > Cs > Y > U > Tb > Be ≥ Ho, and Tl (≤0.06 ng/g). Studying the order of the concentrations of these individual elements, it is apparent that they differ from the concentrations of elements in the wheat flake samples presented by Kiewlitz and Rybicka [3], who determined this order: K (3190 µg/g) > Mg > Na > Ca > Fe ≥ Mn > Zn (14.0 µg/g). Similarly, higher concentrations of Mn, Ca, Mg, K, Fe, and Zn were also measured in other types of cereal flakes [3,33,40]. Flakes with higher proportions of coating layers are generally considered as more valuable food components due to their higher concentrations of elements, especially Fe [2,33]. Cd and Pb contents in non-traditional wheat flakes (Table 1) were determined in the amounts of 5.60–45.2 ng/g and 34.4–57.8 ng/g, respectively, both under the respective maximum values established by [35,37]. To our best knowledge, the concentrations of Be, Ga, Y, Sr, Cs, Ho, Ti, and Tb in wheat flakes have not been well documented. Beryllium was observed in plant samples at a concentration of 25 ng/g; Ga was recorded in plant tissues in a varying content of 0.02–30 µg/g; Y levels in cereals reached 3.5 µg/g, and Ho content in cereals ranged between 1.0 and 5.0 ng/g [41]. The content of Li in plants corresponds to its content in the soil; its concentration in grains was determined as ranging from 0.02 to 13 µg/g [41]. Wheat flakes produced by the thermal treatment process with subsequent flaking contained significantly lower concentrations of these trace elements. The amount of Ti in food of plant origin ranged from 0.13 to 6.7 mg/kg, with the lowest values found in prepared cereals. The average Ti content in wheat grains was 0.9 mg/kg [41], whereas non-traditional wheat flakes contained Ti values that were twice as high. This study detected Ba and Co concentrations in all wheat flakes in the ranges of 218–1050 and 6.11–15.5 ng/g, respectively. Our results are comparable with the findings in [25], where the concentrations of Ba, V, and Sb were determined in cornflakes. Furthermore, non-traditional wheat flakes contained twice as much Sr as cornflakes.

### 3.4. Effect of the Thermal Treatment and Flaking on the Mineral and Trace Element Contents of Wheat Flakes

**Figure 1:**
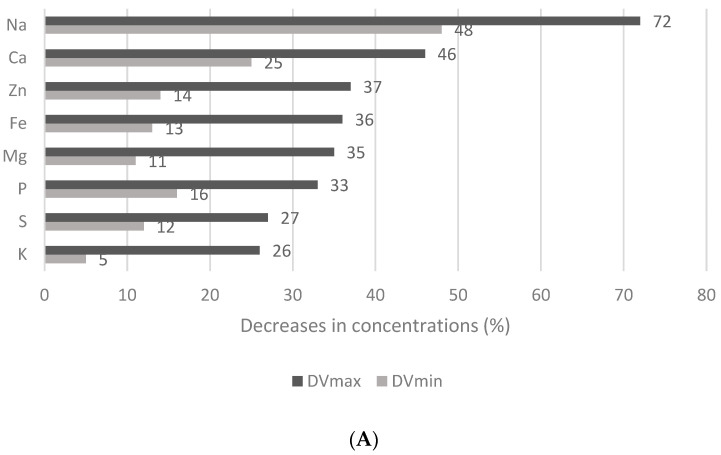

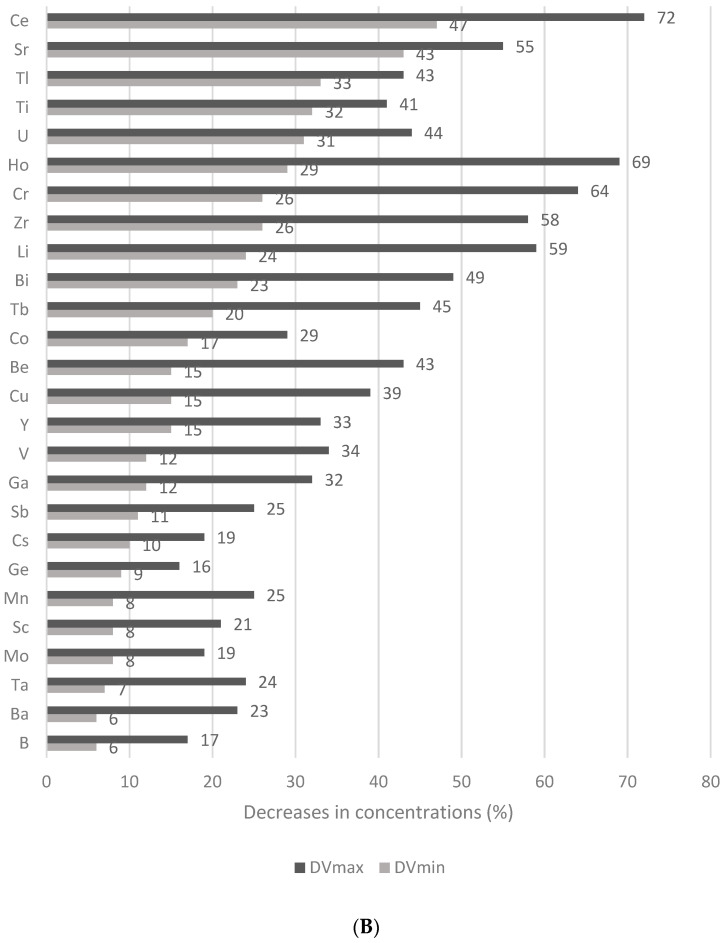

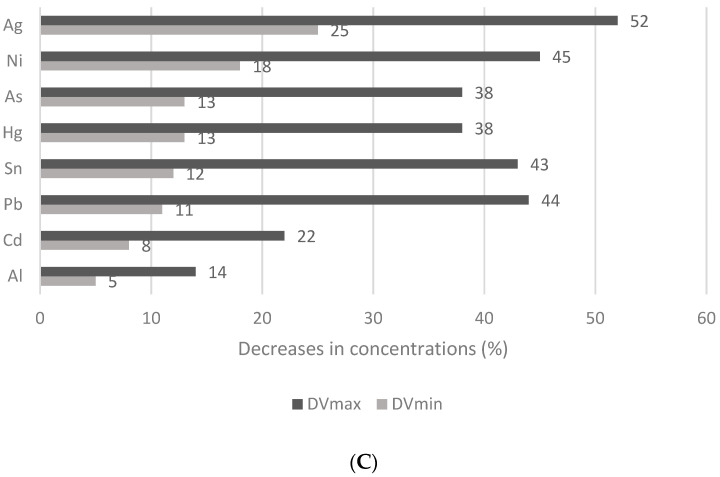
Decreases in the contents (%) of macro and micro elements (**A**), essential and non-essential trace elements (**B**), and toxic trace elements (**C**) during the flake production process.

A total of 43 elements in wheat grains and flakes (Table 1) were measured to assess the value of decrease (DV) in their concentrations after the flake production process. They were divided into three groups: macro and micro elements (Na, Ca, Zn, Fe, Mg, P, S, and K), essential and non-essential trace elements (Sr, Ti, Ho, Zr, Bi, Co, Cu, V, Sb, Ge, Sc, Ta, and B), and toxic trace elements (Ag, Ni, As, Hg, Sn, Pb, Cd, and Al). The minimum and maximum values of the concentration decreases (DV_max_, DV_min_) of each element are presented in Figure 1A–C. The concentrations of macro and micro elements (Figure 1A) declined in this order: Na (48–72%) > Ca > Zn > Fe > Mg > P > S > K (5–26%). The values of essential and non-essential trace elements (Figure 1B) decreased in the following order: Ce (47–72%) > Sr > Tl > Ti > U > Ho > Zr > Li > Bi > Tb > Co ≥ Be ≥ Cu > Y > V > Ga > Sb > Cs > Ge > Mn > Sc ≥ Mo > Ta > Ba > B (6–17%), and finally, the concentrations of toxic trace elements (Figure 1C) declined as follows: Ag (25–52%) > Ni > As > Hg > Sn ≥ Pb > Cd, and Al (5–14%). Only the concentration of Ag dropped by more than 50%. The most significant losses occurred during the hydrothermal treatment and flake production due to the loss of the coating surface layers [5]. The results of this study show that during the thermal treatment and flake production, the highest losses were seen in the concentrations of Na, Sr, Ti, Ho, Zr, Bi, and Ag. Similarly, boiling has been reported to reduce the contents of Ca, Na, K, Mg, Mn, Cu, Fe, and P in rice grains and cooking has stimulated the loss of minerals due to leaching [12].

### 3.5. Estimations of the Dietary Intake of Elements

#### 3.5.1. Estimation of the Dietary Intake of Essential Elements

**Figure 2:**
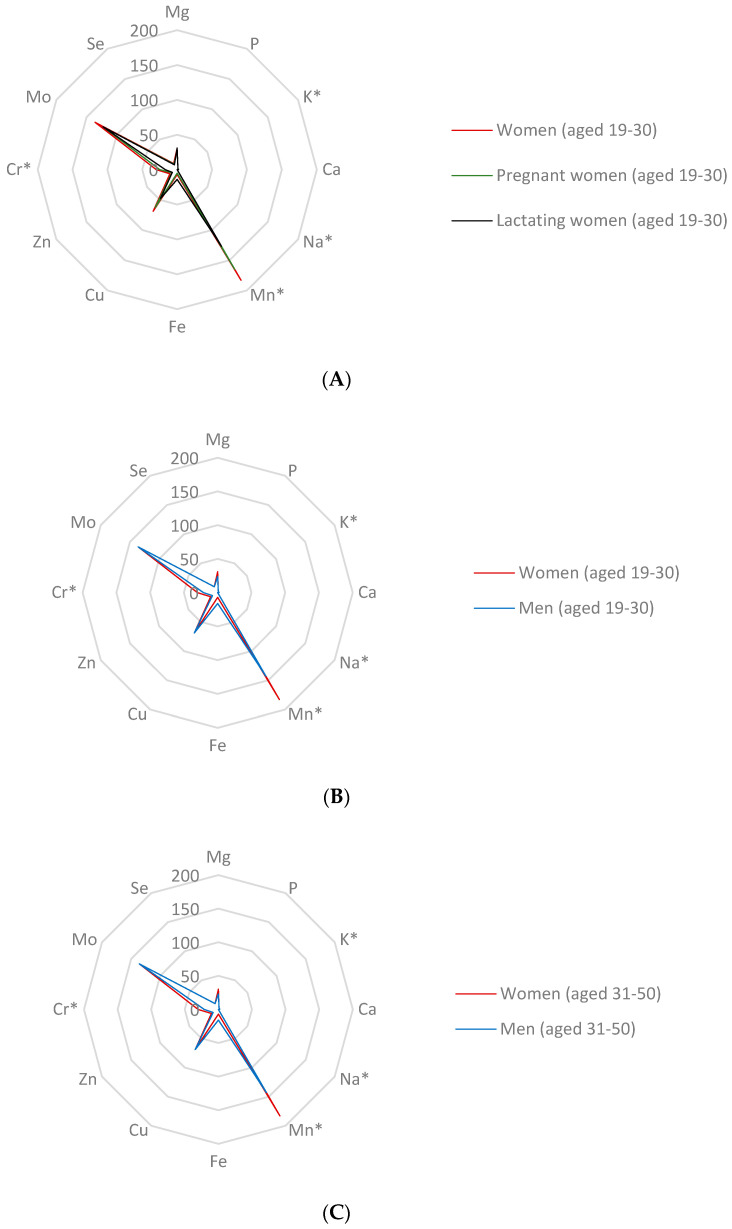

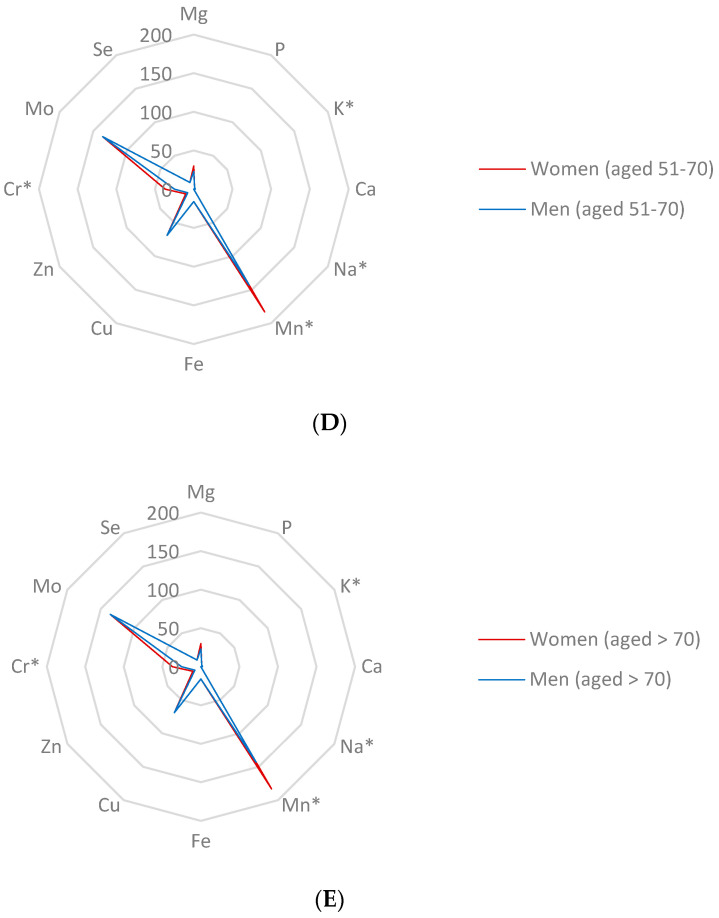
(**A**–**E**). Dietary intake levels (%) of essential mineral and trace elements from non-traditional wheat flakes for individual life stage groups. * Dietary intakes of flakes to AI value for K, Na, Mn, and Cr are followed by an asterisk.

More than two billion people worldwide are affected by mineral and trace element deficiencies. The most vulnerable groups include children under the age of five years and pregnant women. Approximately 33% of women of reproductive age worldwide suffer from anaemia [8]. Apart from anaemia, diet offering insignificant intakes of minerals may lead to nutritional deficiencies, such as osteoporosis and impairments of physical growth [3]. The appropriate reference doses formulated as the RDA or AI values for each individual age group defined by the IOM (Institute of Medicine) are summarized in Table S2. The contribution of the consumption of 100 g of non-traditional wheat flakes to the RDA or AI values was determined with respect to the minimum and maximum values of the total concentrations of essential minerals and trace elements using the reference values for Ca, Cr, Cu, Fe, Mg, Mn, Mo, P, Se, Zn, K, and Na for each life stage group. The results are presented in Tables S4–S7 and Figure 2A–E. According to the Regulation (EU) No 1169/2011 of the European Parliament and of the Council, a food product can be classified as a significant source of minerals when it reaches a minimum of 15% of the nutrient reference value of nutrient supplied by 100 g of the product [42].

As the results show, non-traditional wheat flakes did not contribute significantly to the AI values of Na and K and to the RDA values of Ca, P, Se, and Zn for all age categories and genders. It is essential to obtain less than 0.1% of the AI value of Na to prevent hypertension and 1–2% of the AI of K for men and women of all age groups, as well as pregnant and lactating women. Sodium is an essential mineral involved in regulating blood pressure, participating in the control of the volume and systemic distribution of total body water, influencing nerve and muscle function, and interacting with potassium [8]. However, in excess it can lead to hypertension and to further serious implications, such as congestive heart failure, renal disease, and cirrhosis [43]. 

Potassium is the third most abundant mineral in the human body; it acts as a regulator of the acid–base balance and regulates osmotic pressure, muscle contraction (particularly cardiac muscle), and cell membrane function. It also plays a role in cell metabolism by participating in energy transduction, hormone secretion, and the regulation of protein and glycogen synthesis [8,43].

Calcium is primarily stored in the bones and teeth (99%). The remaining part (1%) is present in extracellular membranes. The element is essential for the muscle contractions, blood coagulation, activation of hormone and enzyme secretions, and nervous system function [8,43]. However, non-traditional wheat flakes are an inefficient Ca source as they provide less than 2% of the RDA for men and women in each category. Ca deficiency can cause poor calcification and defects in dentition. In contrast, elder people are sensitive to an excess of calcium intake due to deteriorated renal functions [8].

Phosphorus is stored in the form of phytic acid in cereals, an antinutrient due to its affinity with Fe, Zn, and Ca [2]. Since approximately 50% of the P content is retained in the bran, cereal grains with these outer layers show higher phosphorus contents. The contribution of flake consumption to the RDA value of P intake is only 2% for each category examined in this study (Figure 2A–E, Tables S4–S7). Phosphorus is employed in cellular energy transport as a part of adenosine triphosphate. Together with calcium salts, P participates in bone stiffening [30,43]. In the past, P deficiency manifested as osteomalacia and rickets, which can be triggered by an inadequate intake of Ca and vitamin D as well. Nevertheless, phosphorus content in the typical diet for adults appears to be sufficient.

The amount of selenium in grains reflects its amounts and bioavailability in soils and vice versa. Selenium binds toxic heavy metals, especially Hg and Cd, and thus contributes to a mitigation of their various pathological effects [43]. Se deficiency affects the expression and function of selenoproteins and is involved in organ and tissue degeneration leading to the onset of Keshan and Kashin–Beck diseases [44]. Low Se intake correlates with several pathologies, such as oxidative stress and inflammatory conditions, *diabetes mellitus*, hepatopathies, and infections [25]. Therefore, sufficient income of Se in the diet is essential, and non-traditional wheat flakes may provide 10% of the RDA of Se for all categories. For pregnant women and women in their lactation period, lower values of 9 and 8% of the RDA for Se were calculated, respectively (Figure 2A). The IOM [18] established an RDA value of 55 µg/day for adults of both genders and 60 µg/day for pregnant and 70 µg/day for lactating women (Table S2). In contrast, the EFSA [44] set the AI of 70 µg/day for adults in general and 85 μg/day for lactating women. Currently, the EFSA [45] has re-evaluated the tolerable upper intake levels (UL) to the value of 255 µg/day for adult men and women, including pregnant and lactating women. 

Non-traditional wheat flakes appear to be an insignificant source of Zn for all examined categories, contributing about 12% of the RDA value for women of all age categories, 9% for pregnant women, 8% for lactating women, and 9% of the RDA value for men of all age groups with a portion size of 100 g. Due to the lack of data regarding nutrient intakes from non-traditional wheat flakes, it is possible to compare the results with the study in [31] explaining that wheat-based food could supply 75–100% of the daily Zn requirement in men and more than 100% in women with the daily portion set between 260 and 370 g per day [31]. Zn is a cofactor for many enzymes and is essential for gene expression and regulation of cellular growth and differentiation [8]. Zn deficiency represents a risk for approximately 33% of the global population due to various factors, such as an inadequate Zn absorption due to the inhibitory effects of phytate and polyphenols in food of plant origin [8,46]. 

In terms of iron, the RDA value for men significantly differs from its value for women in general and for pregnant and lactating women (Table S2). Men of all ages and women aged from 51 to over 70 can obtain up to 16% of their RDA value from flakes. Contributions to the RDA of Fe for women aged 19–50 declines to 7%, for pregnant women to 5%, and finally, for lactating women it reaches 14% (Figure 2A–E). Most Fe is present in erythrocytes as a part of haemoglobin, and its deficiency is associated with anaemia, impaired physical and cognitive performance, and increased maternal and child mortality. In food of plant origin, iron is in an inorganic non-haem form and represents the most abundant type of dietary Fe. Cereal-based food comprises an important dietary source of Fe despite the limited ability of cereals to accumulate it from the soil [33]. Based on the results of this study, non-traditional wheat flakes contribute inconsiderably to reaching the RDA value of Fe for pregnant women and women aged 19–50 with the portion size of 100 g. However, if the portion size was between 260 and 370 g per day, then with the RDA values of 8 and 18 mg/day for men and women, respectively, wheat grain consumption provided more than 100% of the required Fe for adult men and up to 86% of the required Fe for adult women [31].

The consumption of 100 g of non-traditional wheat flakes contributes significantly to the AI values for Cr and Mn, as well as to the RDA values for Cu, Mg, and Mo (Figure 2A–E). It is generally known that biologically active trivalent Cr^3+^ enhances insulin activity, participates in carbohydrate metabolism, and is involved in the metabolism and storage of protein and fats [43,47]. Nevertheless, Cr^6+^ compounds are classified by the International Agency for Research on Cancer (IARC) as carcinogenic to humans [48]. At human dietary exposure levels, Cr absorption is below <10% of the ingested dose and depends on its valence state and ligands. Most of the ingested Cr^6+^ seems to be reduced in the stomach to Cr^3+^, which is poorly bioavailable and with a low ability to enter cells. In contrast, Cr^6+^ can pass through the cellular membrane [48]. Overexposure to Cr^6+^ through its consumption from contaminated food could result in gastrointestinal and neurological effects, abdominal pain, vomiting, cancer, and haemorrhage [47]. Non-traditional wheat flakes provide 37% of the AI value for Cr for women aged over 70. The lowest contribution to the AI value was established as 17% for lactating women (Figure 2A). In this study, individual contributions to the AI value for Cr were calculated from the total Cr amount. The safety of non-traditional flake consumption should be evaluated by the assessment of the Cr^6+^ content in the flakes; importantly, Cr^3+^ has been found to oxidize to Cr^6+^ during the thermal treatment of cereal foods [47]. The maximum acceptable (MAC) daily intake of Cr^6+^ of 0.003 mg/kg bw/day is taken into account to evaluate the safety consumption [49]. Due to the mentioned health risks, the CONTAM Panel of EFSA has not established a safe TDI (tolerable daily intake) level for Cr^6+^. The TDI of naturally occurring Cr^3+^ is 0.3 mg/kg bw/day [48]. 

Magnesium influences bone health through its role in the structure of hydroxyapatite crystals and is also an essential cofactor for enzymes involved in glucose metabolism. Magnesium deficiency is associated with diverse issues including an increased risk of type 2 diabetes [8,10,43,50]. The results of this experiment have confirmed non-traditional wheat flakes as a significant source of Mg for all age groups included in this study (Figure 2A–E, Table S7). The flakes contributed 28–31% of the RDA value for Mg for women aged 19–30 and for lactating and pregnant women and 30% of the RDA of for women aged from 31 to over 70. The contribution of the flakes to the RDA value for men of all ages was 23–24%.

This study has proved that non-traditional wheat flakes are a significant contributor to the RDA of Cu for all age groups. The lowest contribution to the RDA (48%) was evaluated for lactating women aged 19–30; next for pregnant women the contribution to the RDA from flakes was 63% (Figure 2A). Regarding the other age groups (Figure 2B–E), the average intake of flakes represented 69% of the RDA. For the human body, Cu is essential in trace amounts as a constituent of redox enzymes and haemocyanin; it is involved in mitochondrial function, cell metabolism, connective tissue formation, and Fe absorption and storage [8,43]. Cu is found in a variety of foods and its relatively low RDA value indicates that its deficiency is rare [51]. Clinical disorders associated with Cu deficiencies include bone disorders, anaemia, impaired growth and reproductive performance, gastrointestinal disorders, and heart failure. A tolerable upper intake level of 5 mg/day was established for adults; however, due to the lack of data this measure has not been defined for pregnant and lactating women [46]. Recently, the EFSA Scientific Committee has concluded that Cu retention should not occur within an intake of 5 mg/day and established an ADI (acceptable daily intake) of 0.07 mg/kg bw [52].

Molybdenum is an essential trace nutrient that plays a role as a cofactor for sulphite oxidase, xanthine oxidoreductase, and aldehyde oxidase. These enzymes are involved in the metabolism of aromatic aldehydes and the catabolism of amino acids and heterocyclic compounds containing sulphur [43,53]. The RDA values for Mo were suggested as 45 µg/day for all age groups and 50 µg/day for pregnant and lactating women (Table S2). Even though clinical symptoms of Mo deficiency have not been observed, at the request of the European Commission, the EFSA Panel on Dietetic products, Nutrition, and Allergies (NDA) provided a scientific review on dietary reference values for Mo in the European population [53], proposing an AI value for Mo of 65 µg per day for adults. Subsequently, the German-speaking countries [54] set an AI of 50–100 µg/day [53]. The Regulation (EU) No 1169/2011 of the European Parliament and of the Council confirmed a daily nutrients reference value (NRV) for Mo for adults of 50 µg/g [42]. This study has confirmed that non-traditional wheat flakes provided a contribution to the RDA of up to 136% for all age groups and 123% for pregnant and lactating women, which is in alignment with the fact that cereals and cereal-based products account for the majority of the dietary Mo intake in adults [53].

Concerning manganese intakes, there are some similarities to Mo. The AI values for Mn were set as follows: 2.3 and 1.8 mg/day for men and women aged 19 to over 70, respectively, and 2.0 and 2.6 mg/day for pregnant and lactating women, respectively (Table S2). The EFSA Panel on Dietetic products, Nutrition, and Allergies (NDA) provided a scientific recommendation on dietary reference values for Mn in the European population [55] and suggested an increased AI value for adults of 2–3 mg/day. The Regulation (EU) No 1169/2011 of the European Parliament and of the Council has affirmed a daily nutrients reference value (NRV) of Mn for adults of 2.0 mg/g [42]. Based on the results of this study and dietary intakes suggested by the Institute of Medicine [19], non-traditional wheat flakes contribute significantly to the AI values of Mn in all age groups. A 100 g portion of the flakes supplies 183 and 143% of the AI of Mn for men and women of all ages, respectively, 165% of the AI for pregnant women, and 127% of the AI for lactating women (Figure 2A–E). Since cereal-based products are rich Mn sources [55], a specific Mn deficiency syndrome has not been described yet. Mn is an essential nutrient in humans and plays roles in bone mineralization, regulation of protein and energy metabolism, and protection of cells against damaging free radicals [8]. It is a component of metalloenzymes and is involved in amino acid, lipid, and carbohydrate metabolism [43,55]. Its role in enzyme activation and glycoprotein and proteoglycan synthesis has also been recognized [8]. 

In summary, this experiment has shown that non-traditional wheat flakes contribute to the RDA or AI values of particular minerals and trace elements in the following order: for men of all ages—Mn (up to 143%) > Mo > Cu > Mg ≥ Cr > Fe > Se > Zn > P ≥ Ca ≥ K > Na (less than 0.1%); for women—Mn (up to 183%) > Mo > Cu > Cr ≥ Mg > Fe > Zn > Se > P ≥ Ca ≥ K > Na (below 0.1%); for pregnant women aged 19–30—Mn (up to 165%) > Mo > Cu > Mg > Cr > Zn ≥ Se > Fe > P ≥ Ca > K > Na (below 0.1%); and finally, for lactating women—Mn (up to 127%) > Mo > Cu > Mg > Cr > Fe > Zn ≥ Se > P ≥ Ca > K > Na (below 0.1%) (Figure 2A–E, Tables S4–S7).

#### 3.5.2. Estimation of the Dietary Intake of Toxic Elements

Toxic elements could harm living organisms, especially considering their tendency to accumulate over time, their poor biodegradability, and their long biological half-lives [56]. Based on their occurrence, toxicity, and potential harmful health effects, As, Cd, Pb, and Hg are classified in the top ten compounds of the Substance Priority List (SPL) created by the Agency for Toxic Substances and Disease Registry [57]. 

**Figure 3:**
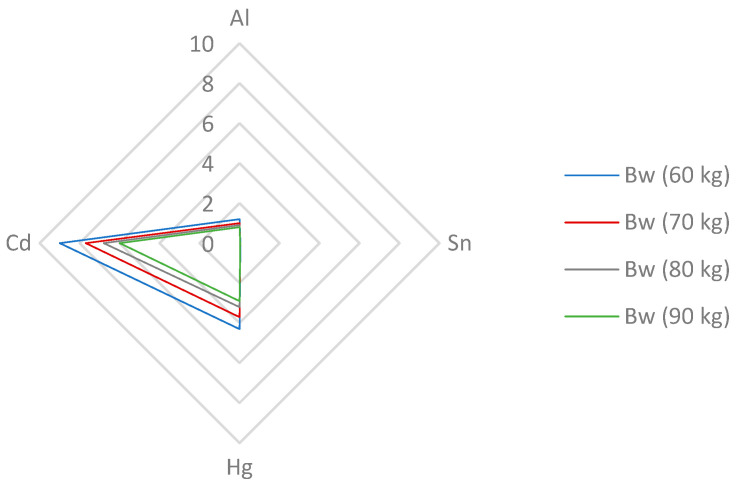
Dietary intake levels (%) of toxic trace elements from non-traditional wheat flakes with respect to body weight.

Even though Cd absorption from the diet is relatively low (3–5%), it must be monitored as Cd has a very long biological half-life of up to 30 years and may be retained in the kidneys and liver causing dysfunctions [33,58]. The provisional tolerable monthly intake (PTMI) of 25 µg/kg bw was established by the Joint FAO/WHO Expert Committee on Food Additives (Table S2) [24]. The suggested PTMI value corresponds to a weekly intake of 5.8 µg/kg bw [59]. As can be seen from the results of this study (Figure 3, Tables S7 and S8), the estimated contribution of wheat flakes to the PTMI value does not exceeded 6 and 9% for persons weighing 60 and 90 kg, respectively. With a 100 g portion of non-traditional wheat flakes, the daily intake of Cd is only 0.56–4.52 µg (Table S8). In the study determining toxic elements in wheat grains, a contribution of 13% for wheat-based products to the Cd intake was found; however, it must be emphasized that these results were calculated using the previous PTWI value, which was 7 µg of Cd per kg [33,58]. Similar results were obtained for gluten-free products and rice grains for celiac patients [25,43]. Since the Joint FAO/WHO Expert Committee on Food Additives (JECFA) re-evaluated the initially suggested provisional tolerable weekly intake (PTWI) from 7 to 25 µg of Cd per kg of bw in 2010, the CONTAM Panel of the EFSA had to review the appropriateness of the tolerable weekly intake (TWI) value of 2.5 μg/kg bw of Cd and whether it should be maintained [58,59]. The TWI for Cd was established as being between 1.9 and 3.0 µg/g per week based on the average dietary exposure to Cd for adults in European countries. The main food categories contributing to the TWI of Cd include grain products (27%) and vegetables (16%) [33,36]. 

Mercury toxicity depends on its chemical form; its methylmercury form is described as the most toxic [56,60]. The symptoms of Hg poisoning include neurological, renal, cardiovascular, and reproductive issues [43]. To estimate the provisional tolerable weekly (PTWI) intake (Table S2), a dietary exposure of 4 µg/kg bw was defined by the Joint FAO/WHO Expert Committee on Food Additives (JECFA) in 2011 [22]. A very low daily Hg intake of 1.13–1.48 µg was calculated from the portion of non-traditional wheat flakes (Table S8). The results of this study present the contributions of wheat flakes to the PTWI of Hg as being from 3.0 to 4.3% for men and women weighing 90 and 60 kg, respectively (Figure 3). A similar contribution of about 4.3% to the PTWI was found in the study analysing the toxic elements in wheat grains [30]. Taking into account other studies, the daily Hg intake from 100 g of cornflakes was found to be 1.83 µg, corresponding to a contribution of 2.5% to the PTWI of Hg of 5 µg/kg of bw [25]. Similarly to Cd, the EFSA readjusted Hg safety levels and confirmed the appropriateness of the 4 μg/kg bw PTWI of Hg set by the Joint FAO/WHO Expert Committee on Food Additives (JECFA). In accordance with the JECFA, the CONTAM Panel established a tolerable weekly intake (TWI) for Hg of 4 μg/kg bw [60].

The main source of aluminium is the diet, specifically cereals, cereal-based products, and vegetables [61]. Aluminium has been associated with Alzheimer’s disease and other neurodegenerative diseases, such as Parkinson’s disease and amyotrophic lateral sclerosis, and shown to be involved in neurotoxicity in patients undergoing dialysis [61,62]. In 2011, the Joint FAO/WHO Expert Committee on Food Additives (JECFA) re-evaluated Al health risks and readjusted and updated its PTWI value to 2 mg/kg bw (Table S2) [23]. As can be seen in Figure 3, the levels of dietary intake of non-traditional wheat flakes to the PTWI of Al varied from 0.8 to 1.2% for adults weighing 90 and 60 kg, respectively. Higher contributions to the PTWI of Al were calculated for teff grains, where a 100 g portion contributed 6 and 7% of the PTWI for adults weighing 80 and 65 kg, respectively [63]. A 71 g portion of rice grains contributed up to 17.9% of the PTWI [43]. In the EU, the average dietary exposure from water and food showed large variations between the countries, ranging from 1.6 to 13 mg of Al per day and corresponding to 0.2 to 1.5 mg/kg bw per week in a 60 kg adult. Considering the cumulative nature of aluminium, the ACF Panel (the Panel on Food Additives, Flavorings, Processing Aids, and Food Contact Materials) has established a tolerable weekly intake (TWI) for Al of 1 mg/kg bw per week [61].

Concerning tin, the Joint FAO/WHO Expert Committee on Food Additives (JECFA) readjusted the previously established provisional maximum tolerable daily intake (PMTDI) of 2 mg/kg bw to a provisional tolerable weekly intake (PTWI) of 14 mg/kg bw; this equivalent to 120 mg/day for a 60 kg adult (Table S2). Inorganic Sn compounds generally have only low toxicity in animals due to their limited absorption from the gastrointestinal tract [21]. The main dietary source of inorganic tin is food packaged in unlacquered or partially lacquered Sn-plated cans; what is more, inorganic Sn migration from tinplate to food is greater in highly acidic foods [21,62]. In this study, non-traditional wheat flakes contributed less than 0.1% of the PTWI for Sn for each examined weight category (Figure 3, Table S7). Similar results were obtained in the study presented in [62]. However, data examining Sn intake in the EU countries is scarce and the Scientific Panel on Dietetic Products, Nutrition and Allergies of the EFSA considered the available data insufficient to derive a tolerable upper intake value. Regulatory limits for the Sn concentration have been established for canned food and beverages; these values are 200 and 100 mg/kg, respectively [64]. Furthermore, a maximum limit of 50 mg/kg for the inorganic Sn form is defined only for processed cereal-based food for infants and young children [65].

Lead has been associated with a wide range of serious health issues, including neurological and behavioural problems, increased mortality (mainly due to cardiovascular diseases), impaired renal function, hypertension, and impaired fertility. The average Pb dietary exposure for adults in the EU ranged from 0.36 to 1.24 µg/kg bw [66,67,68]. The CONTAM Panel concluded that the current PTWI value of 25 μg/kg bw was no longer appropriate. For most Europeans, cereals, vegetables, and tap water were considered as the main sources of Pb intake [68]. This study shows that a 100 g portion of non-traditional wheat flakes provides a daily Pb intake of 3.4–5.8 µg (Table S9), which is lower than the daily Pb intake from wheat grains grown in Serbia, which are associated with a daily intake of about 36 µg [30].

The toxicity of As depends on its chemical form and solubility; As^3+^ is generally more toxic than As^5+^. The main serious issues associated with a long-term ingestion of inorganic arsenic include cancer, skin lesions, cardiovascular disease, neurotoxicity, and diabetes. The FAO/WHO [69] reported the average dietary exposure to inorganic As in the USA and various European and Asian countries as ranging from 0.1 to 3.0 μg/kg bw per day. Despite these facts, the EFSA Committee withdrew the PTWI of 15 μg/kg bw (2.1 μg/kg bw per day) as it was within the limits of the BMDL0.5 value and thus no longer appropriate [22]. The CONTAM Panel came to the same decision [70]. The EFSA has reported that the average dietary exposure for the adult population varies from 0.09 to 0.38 µg/kg bw. Grain-based processed products, mainly wheat bread and rolls, have been considered as the main contributors [69]. Examining non-traditional wheat flakes (Table S9), a 100 g daily portion provides only 586–1090 ng of As, which is below the limits published in [70]. When compared with rice grains, a daily intake of 14 µg corresponded to approx. 12% of the previously defined PTWI value; the milling of rice grains reduced the As content to about 8% of the PTWI [30]. 

Nickel may serve as a cofactor or structural component of specific metalloenzymes with various functions, including hydrolysis and redox reactions and gene expression [19]. Oral exposure to Ni affects the neurological, gastrointestinal, and immune systems and may trigger haematological issues; an acute exposure to Ni causes gastrointestinal and neurological symptoms [71]. The Institute of Medicine estimates a tolerable upper intake level of 1 mg/day [19]. As Table S9 represents, a 100 g portion of the examined non-traditional flakes responds to a daily Ni intake of up to 20.8 µg/day, which proves the flakes as safe. The CONTAM Panel adjusted the tolerable daily intake (TDI) from 2.8 µg/kg bw to 13 µg/kg bw [72]. Using this TDI value we can establish that the contribution is a maximum of 2.7% from one portion of wheat flakes. Akinyele and Shokunbi [73] considered a daily portion of cereals of up to 370 g and an average Ni content in cereals of 0.09 mg/kg, which corresponds to a Ni intake of 7.13 µg/day. Generally, grain and grain-based products, non-alcoholic beverages, sugar and confectionery, and legumes were observed as the main contributors to dietary Ni within different dietary surveys and examined age groups [71].

Soluble silver compounds are absorbed more readily than metallic or insoluble Ag substances and thus pose health risks. Acute symptoms of overexposure to Ag include diarrhoea, decreased blood pressure, respiration issues, and stomach irritation [74]. A chronic exposure to Ag causes argyria, and Ag can be deposited in various organs. Ag that remains in the body is ultimately oxidized to the insoluble Ag_2_S form that is responsible for the skin darkening in argyria [75]. Soluble Ag compounds can also accumulate in small amounts in the brain and muscles. Ag is not carcinogenic [74]. However, it has been added to the Substance Priority List for toxic substances [57]. The estimated daily dietary intake is 0.91 µg/day, with the main contribution coming from cereals, fruits, and vegetables according to [62]. If wheat flakes contain 0.89–5.07 ng of Ag per 1 g (Table 2), a portion of 100 g provides a maximum dietary intake of 507 ng/day. However, a tolerable upper intake level or provisional tolerable daily or weekly intake levels for Ag have not been established yet.

#### 3.5.3. Estimation of the Dietary Intake of Other Elements

The appropriate daily intakes of other elements are presented in Table S9. Sulphur is a constituent of many proteins due to its presence in amino acids, such as cysteine and methionine, and is present in biotin and vitamin B_1_ [43,76]. The guidelines for dietary sulphur intake have not been established.

Cobalt is an essential trace element incorporated in some metalloproteins and vitamin B_12_; it plays a key role in the normal functioning of the brain and nervous system and in the formation of the blood [25]. Excessive Co retention in the body over time could be toxic, especially harming the liver. The Scientific Committee of the EFSA concluded that no Co retention is expected with an intake of up to 5 mg per day and established an acceptable daily intake (ADI) of 70 µg/kg bw for adults. The levels of different food categories’ contributions to dietary Co exposure in various age groups were established in the following order: grains and grain-based products > fruit products > meat products > vegetable products > coffee, cocoa, tea, and herb infusions [52]. 

Oral exposure to antimony results in burning stomach pains, colic, nausea, and vomiting; additionally, Sb toxicity can negatively affect the liver, skin, and the respiratory and cardiovascular systems. In 2003, the World Health Organization (WHO) determined a total dietary intake (TDI) for Sb of 6 µg/kg bw from drinking water [77,78], indicating that the population is generally exposed to low amounts of Sb [79]. 

Exposure to high doses of soluble barium compounds causes a number of issues including cardiogram abnormalities, tachycardia, hypertension, hypotension, and muscle weakness. Kidney disorders are associated with long-term Ba exposures. The TDI value for Ba was established as 0.2 mg/kg bw [77,80].

The daily intake of strontium by adults is estimated to be approximately 4 mg. The main sources of Sr include leafy vegetables, grains, dairy products, and drinking water. Sr is poorly absorbed by the body, with an absorption level of up to 30%. Most of the absorbed element is deposited in the bones but it can also bind to calcium transporting proteins [81,82]. Currently, there is no standard limit for Sr in food. This study shows that the Sr intake ranges between 75.6 and 143 µg/day in a 100 g portion of non-traditional wheat flakes (Table S9).

Vanadium has been proven to be of minor importance for human health concerning its nutritional value. Therefore, nutritional requirements or intake recommendations for V have not been established. Nonetheless, high V concentrations can cause irreversible damage to the kidneys and some of its chemical forms may trigger gastrointestinal problems, such as diarrhoea, vomiting, intestinal inflammation, and a characteristic green tongue [77]. On the other hand, V deficiency could result in hypothyroidism and may exacerbate the effects of pre-existing iodine deficiency. A reference dose (RfD) of 7 µg/kg bw per day was derived by the EPA (Environmental Protection Agency of the United States) based on gastrointestinal disturbances observed in human studies [83]. The V intake from food is estimated as 10–20 µg/day. Whole grains generally contain vanadium in amounts of 5–30 µg/kg [84]. Considering a 100 g portion of wheat flakes, the daily intake of V ranged from 1.74 to 11.2 µg/day (Table S9), which is comparable with the EPA data for its intake from food. These values are at least three orders of magnitude below the lowest doses reported to cause health issues.

Therapeutic lithium compounds have been widely used in psychopharmacology, particularly in the treatment of bipolar disorder [85]. Li may negatively influence renal functions, with the most common disorder being nephrogenic *diabetes insipidus*. Li can also affect thyroid functions; asymptomatic hypothyroidism has been observed in patients being treated with Li [77]. The estimations for daily Li intake vary a lot, from several units to several thousands of µg per day. Although Li is not officially considered as a micronutrient, Schrauzer [86] has suggested provisional recommended intakes of 1000 µg/day for a 70 kg adult (14.3 µg/kg bw). However, these estimations do not reflect individual differences and cannot be formally used in dietary practice. In Europe, Li intake is approximately 10.7 µg/day [85]. Considering a 100 g portion of wheat flakes, Li daily intake is only 1.47–4.36 µg/day (Table S9).

Even though boron is not classified as an essential nutrient and has not been associated with any specific biochemical functions, it could influence the metabolism of other nutrients, particularly Ca, and thus may have a beneficial effect on bone calcification and maintenance. The recommended B intakes have not yet been established; however, its average intake from food is estimated to be 1.5 mg/day in adults. Its main dietary sources include fruits, leafy vegetables, nuts, and legumes [84,87,88]. The IOM [19] stated its dietary intake as ranging between 0.87 and 1.35 mg/day for adults and between 1.05 and 1.08 mg/day for pregnant women. For lactating women, its intake reaches 1.27 mg/day [89]. The WHO first established an acceptable safe range for the intake of B of 1–13 mg/day [90] but later changed it to a total daily intake of 0.4 mg/kg bw [91]. The IOM [19] suggested a tolerable upper intake level of 20 mg/day; the EFSA established an upper intake level for total B intake based on bw that equals about 10 mg/day for adults [87]. This study has confirmed B concentrations in the range 217–341 ng/g that correspond to a daily intake of B of 21.7–34.1 µg within a 100 g portion of flakes (Table S9), which is below the limit. As the total daily intake has been set to 0.4 mg/kg bw, th 100 g portion of wheat flakes contributes less than 0.15% of the total of this value for a person weighing 60 kg. Evidently, non-traditional wheat flakes do not significantly contribute to B daily intake. 

Thallium is classified as a cumulative poison, and its salts may cause a wide spectrum of health issues. Acute poisoning is usually accompanied by gastrointestinal symptoms and chronic exposures result in neurological disorders (motor and sensory changes), encephalopathy, polyneuritis, tachycardia, and degenerative changes of the heart, liver, and kidneys [77,92,93,94]. What is more, exposure to Tl during pregnancy influences mitochondrial DNA in newborn babies [94]. Naturally, Tl occurs as a trace element in many minerals and its concentration in the soil correlates with the geological composition. Kabata-Pendias [41] reported Tl contents ranging from 30 to 300 µg/kg in grains and cereals, leafy vegetables, beans, and potatoes. This study determined significantly lower Tl concentrations of 4–6 ng/100 g in non-traditional flakes (Table S9). Nevertheless, limited toxicological data and naturally low Tl amounts in the environment complicate the measurement of its concentrations in food. Therefore, it is important to collect more data on its presence in the diet. This problem has been addressed by the EFSA as a part of the EU-FORA program, incorporating the observation of Tl together with that of Te and other rare earth elements [94]. To date, recommended intakes are unavailable for these elements.

### 3.6. Effect of the In Vitro Digestibility on the Mineral and Trace Element Retention in Wheat Flakes

The intake levels of elements calculated from their concentrations in non-traditional wheat flakes are overestimated as the digestion processes in the gastrointestinal tract may modify their bioaccessibilities and retentions from the matrix of the flakes. This study has assumed that the undigested flake portion continues through the digestive tract to the large intestine and evaluated the effect of the *in vitro* digestibility of flakes on their mineral and trace element contents. Therefore, this study includes the analysis of the undigested portion of flakes with the objective to determine the concentration of the elements and calculate the retention factors (RFs, %) for the individual elements. To calculate the RF value for each element (RF_max_, RF_min_), the concentrations of individual elements were assessed in both the native (Table 1) and undigested portions of the wheat flakes (Table 2). 

The RF value indicates the amount of the determined analyte remaining in the undigested portion after the digestion process. The higher RF value, the higher amount of the analyte remaining in the matrix of the undigested part of the sample and thus not available for absorption in the digestive tract. Conversely, analytes with low RF values could be potentially more accessible for absorption in the tract [13].

**Figure 4:**
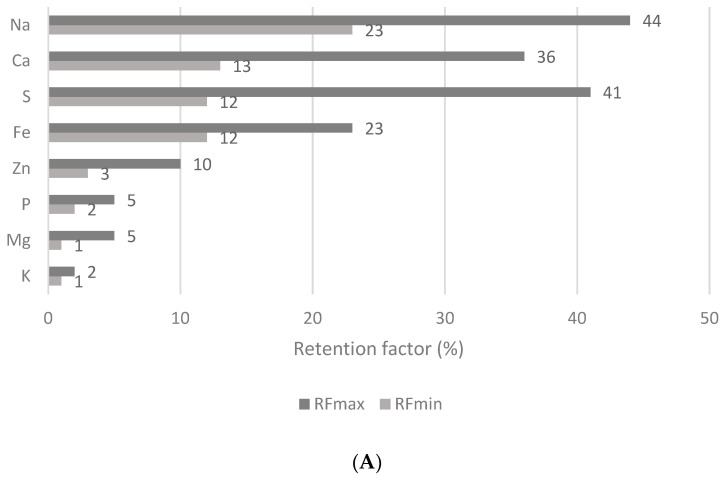

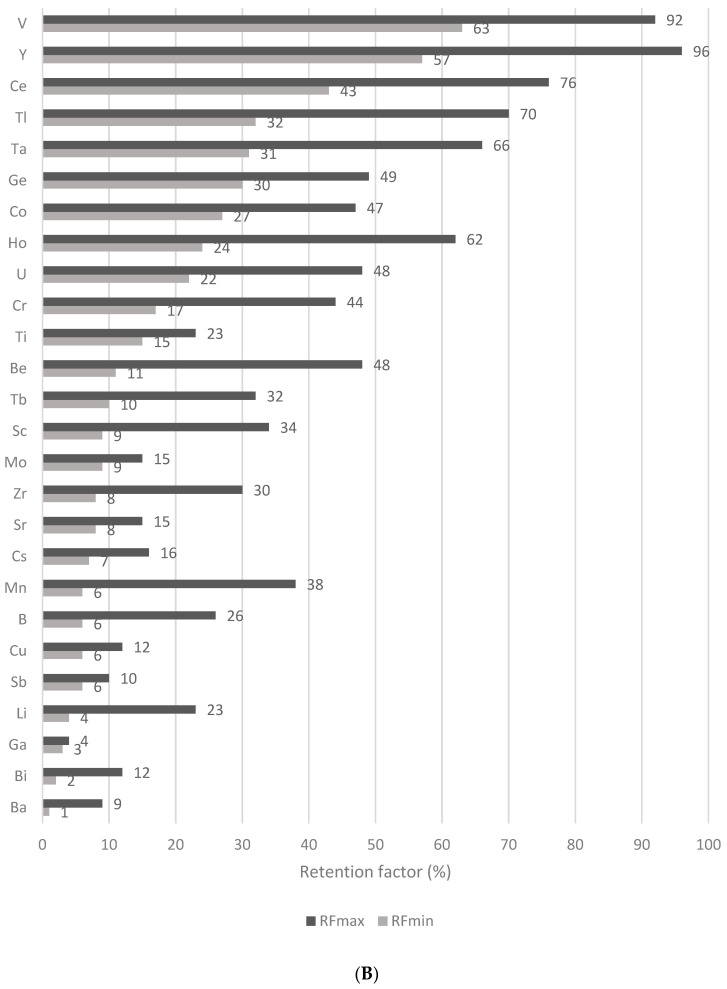

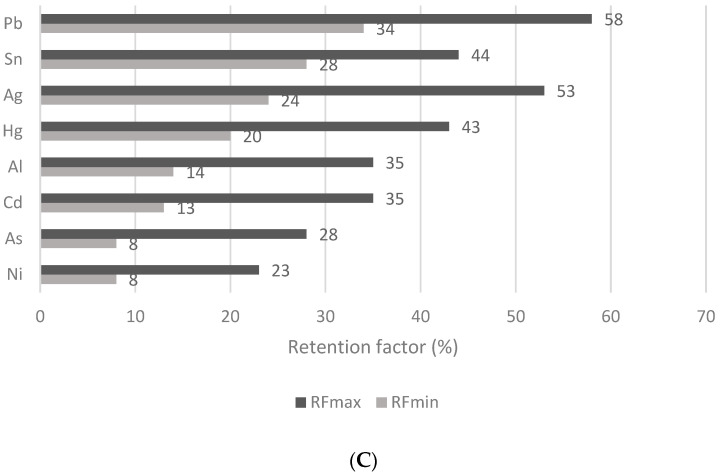
Remaining proportions (%) of macro and micro elements (**A**), essential and non-essential trace elements (**B**), and toxic trace elements (**C**) after simulation of *in vitro* digestion of non-traditional wheat flakes.

The results were calculated as minimum and maximum RF values (RF_min_, RF_max_) for each analyte in wheat flake samples and are depicted in Figure 4A–C. Concerning the group of macro and micro elements (Figure 4A), Na, S, and Ca were retained in the sample matrix at the highest proportions, with RF_max_ values of 44, 41, and 36%, respectively. In contrast, K was retained in the original matrix of the flake at only 2%, whereas only 5% of Mg was retained. In terms of essential and non-essential trace elements (Figure 4B), the highest RF_max_ values were calculated for Y, V, Ce, Tl, Ta, and Ho. The RP values were above 62%; in the cases of Y and V they were even more than 92%. It seems that Ga, Ba, Sb, Bi, Cu, Sr, and Mo are the least retained in the matrices of the wheat flake samples, with RF_max_ values in the range of 4–15%. Regarding toxic trace elements (Figure 4C), the highest RF_max_ value of 58% was calculated for Pb, followed by Ag with an RF_max_ of 53%. On the other hand, the lowest RF_max_ value of 23% was observed for Ni. These RF_max_ values define the order in which the elements are more easily released from the sample matrix during *in vitro* digestion to be potentially absorbed at greater levels by the human tract in the following order: K (2%) > Ga (4%) > Mg and P (5%) > Ba (9%) > Zn and Sb (10%) > Bi and Cu (12%) > Sr and Mo (15%) > Cs (16%) > Fe, Li, Ti, and Ni (23%) > B (26%) > As (28%) > Zr (30%). This study shows that some elements might be retained in the undigested residue of the wheat flakes and could enter the large intestine. What is more, many analytes were released at less than 50% of their total amounts, and their RF_max_ values are as follows: Y (96%) > V (92%) > Ce (76%) > Tl (70%) > Ta (66%) > Ho (62%) > Pb (58%) > Ag (53%).

*In vitro* methods have been designed as an alternative to *in vivo* methods to estimate mineral bioavailability. These methods are based on the simulation of gastrointestinal digestion involving two or three digestion steps simulating oral (optional), gastric and intestinal digestion with possible variations based on the amount and type of the enzymes involved [13,95] and factors including pH and duration of digestion [7,8,31]. Limited data discussing the remaining proportions of mineral and trace elements from non-traditional wheat flakes complicates the comparison of the results obtained in this unique study with other findings. The retention of minerals and trace elements and their bioacessibilities can be influenced not only by the types of cereal grains and their varieties [12] but also by the technological parameters of the wheat flake production, such as soaking and cooking in water, heating and overheating, and high-pressure techniques [8]. For example, pressure cooking decreased the bioaccessibility of Zn by 57–63% and improved the bioaccessibility of Ca and Cu by 13–52% [8]. This increase may stem from the elimination of mineral binders and the increase in mineral enhancers, such as organic acids. Similarly, phytate in breakfast flakes [2] has been confirmed as the main inhibitor of Fe and Zn absorption in the tract [4,6,31]. Moreover, the formation of insoluble Fe complexes with polyphenols and antinutrients and their precipitation as hydroxides also explains the low Fe bioaccessibility [6]. As can be seen from the results (Figure 4A), only 3–10 and 12–23% of Zn and Fe, respectively, are retained in the undigested part of non-traditional wheat flakes. Theoretically, Zn and Fe can be absorbed by the human digestive tract at levels of more than 90–97 and 77–88%, respectively. Therefore, it may be assumed that the thermal treatment process and subsequent flaking process facilitates the release of Zn and Fe from the matrix of the wheat grains, especially from complexes with phytic acid. Different results were obtained analysing the flakes proceeded from sprouted wheat grains with the bioaccessibilities of Fe and Zn at only 3–5% due to their chelation by phytic acid [2]. Phytate activity is stimulated during soaking and deactivated at temperatures of more than 80 °C [12]. The RF value of Ca in non-traditional wheat flakes ranged from 13 to 36% (Figure 4A), which may correspond to its availability for absorption in the digestive tract of 64–87%. Interestingly, the inclusion of citric acid enhanced the bioaccessibility of Ca, Zn, and Fe, whereas ascorbic acid has not shown the same effect [9]. The RF values for Mg (Figure 4A) and Mn (Figure 4B) show that Mg and Mn could be absorbed by the gastrointestinal tract at levels of 95–99% and 62–94%, respectively. These results are in concordance with the study in [6], which detects mineral bioaccessibility after oat flour digestion. The bioaccessibility of Mn has been attributed to its high solubility in the intestinal tract. Even though Mn is bound to proteins, lipids, and fibres, it also exists in a free form or in soluble complexes with phytochelatins, hydrolysable lipids, soluble proteins, or sugar compounds [6]. This study evaluates the RF value for Tl in wheat flakes as being between 32 and 70% (Figure 4B), showing that 30 to 68% of it can be potentially absorbed. Cvjetko et al. [93] reported that Tl is almost completely absorbed by the mucosa (80–100%), enabling its fast distribution from the blood to tissues.

Concerning toxic elements (Figure 4C), Ni, As, and Cd appear to be the most released elements from the wheat flakes matrix; however, Pb, Sn, and Ag are significantly retained in the undigested part of the flakes. The RF values for Ni, As, and Cd were established as being between 8 and 23, 8 and 28, and 13 and 35%, respectively. This means that 87% of Cd could be absorbed by gastrointestinal tract. Similar results were demonstrated in [56], which reported the highest bioaccessibility for Cd, followed by As and Pb. This may be related to the low pH in the tract increasing the solubility of metals [96]. In addition, the bioaccessibilities for toxic elements (especially As) from raw rice grains varied depending on the country of origin [56]. Therefore, the second part of the EU-FORA program placed special attention on the impact of the food matrix on the bioaccessibility and bioavailability of heavy metals and metalloids. With the increasing variety of available foods, it is fundamental to understand about the effectiveness of the absorption of contaminants. This is why a database of the bioaccessibilities and bioavailabilities of Cd, Pb, Hg, and As in different food matrices was created [97].

The recommendations for the human diet should include foods with low retention factors for essential mineral and trace elements, as these elements are involved in vital functions connected with mineral metabolism. Concerning safety and risk assessment, it is necessary to monitor toxic elements and their release from the food matrix during digestion and their retention in the undigested part of food that can reach as far as the large intestine. Control and regulation of allowable and maximum permitted concentrations of toxic heavy metals in food are performed by the Food and Agriculture Organization and World Health Organization, European Commission, and other regulatory bodies [32]. 

### 3.7. Effect of Flake Production and In Vitro Digestion on the Metal Pollution Index

The discharge of industrial effluent containing heavy metals onto agricultural fields has degraded soil quality and affected food quality. Food and water consumption are the main pathways for heavy metals entering the body [98]. Apart from plant species, pH, particle size, soil cation exchange capacity, redox potential, seasons, and root exudation influence the uptake and accumulation of heavy metals in plants [26,98]. Prolonged consumption of food contaminated with heavy metals may disrupt numerous metabolic processes and may subsequently lead to various kidney, liver, bone, and neural disorders, in addition to the impediment of immunological responses, intrauterine growth retardation, and numerous types of cancer [26,98]. To prevent this, the metal pollution index is one of the parameters used to monitor the overall heavy metal concentrations in food [25,26]. Although Co, Cu, Fe, Mn, Zn, Mo, and Se are metals essential for living organisms, they contribute to health issues when ingested in amounts higher than the safe limit. Further metals, including As, Ni, Cd, Cr, Pb, Hg, Tl, and Co, may cause health problems even at low concentrations [99]. Drinking water, vegetables, rice, and milk are listed as having the highest MPI values [98,99]. Furthermore, the consumption of cereal grains and cereal-based products is another significant pathway of heavy metal exposure and a risk to human health.

**Figure 5:**
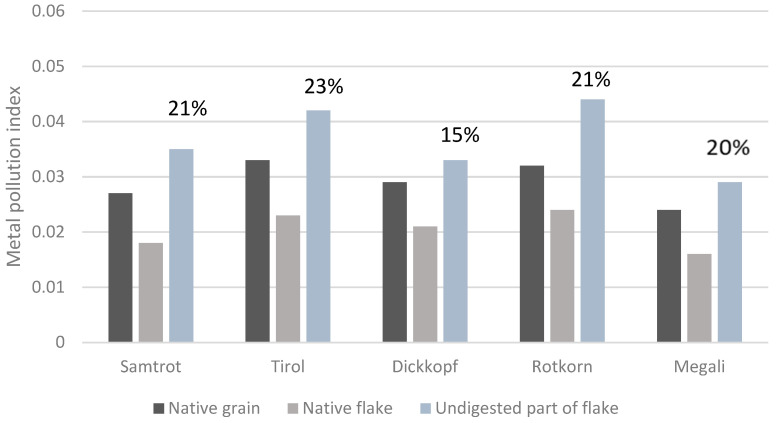
Metal pollution index of native grains and native and undigested flakes. RP_MPI_—the remaining part of the metal pollution index after the digestion of the flakes is expressed in % (above the third column for each sample).

Therefore, this study provides the MPI values (Figure 5, Table S10) not only for grains and flakes but also for the undigested parts of the flakes in order to evaluate which part of the MPI value corresponds to the particular parts of the native flakes of non-traditional wheat flake matrix. Among individual non-traditional wheat grains, the Tirol and Rotkorn varieties exhibited the highest MPI values of 0.033 and 0.032, respectively, followed by Dickkopf, Samtrot, and Megali. Lower MPI values ranging between 0.016 and 0.024 were found for non-traditional wheat flakes produced from grains using thermal treatment followed by flaking. The lower MPI values for flakes compared with grains could stem from the elements leaching into the water during the hydrothermal treatment. The MPI index for the undigested part of the wheat flakes reached values of 0.029–0.044. Since 1 g of the native fraction of flakes does not form 1 g of the undigested part after the *in vitro* simulation of digestion, the digestibility values varied from 88.7 to 90.5%. The RP_MPI_ values in % were calculated to evaluate which part of the MPI value is retained in the undigested part of the flakes. The results show that 15–25% of the MPI value assessed for native flakes remains in the undigested portion, which is in alignment with the results reported in [25], which examined gluten-free shortbread and local bread that had MPI values of 0.44 and 0.42, respectively. Noticeably different results were obtained for wheat grains harvested in an industrial area in India, with MPI values of 12.8–24.4% based on the concentrations of Zn, Ni, Cu, Cd, and Cr [99].

## 4. Conclusions

This study provides data on the mineral and trace element contents in non-traditional wheat grains and flakes and their undigested portions. It also investigates declines in element concentrations after the flaking processes. Furthermore, it evaluates the retention factors of the individual elements after the *in vitro* digestion process. This unique study estimates dietary intakes of essential and toxic elements based on a 100 g portion of flakes for individual life stage groups and assesses the metal pollution index. The mineral and trace element contents of non-traditional wheat flakes were established as being in the following order: Mg (up to 967 µg/g) > K > Ca > P > Mn > Fe > Zn > S ≥ Cu > Na > Ti ≥ Al (up to 2.01 µg/g) and Sr (up to 1430 ng/g) > Ba > Mo > Sn > B > Ni > V > Cr > Sc > Pb > Se > Cd > Li > Zr > Co > Ga ≥ Hg > As > Ta > Ce > Sb > Ag ≥ Ge > Bi > Cs > Y > U > Tb > Be ≥ Ho, and Tl (≤0.06 ng/g). Considering the effect of the flake production process, the highest decreases were observed in the concentrations of Na (48–72%), Ce (47–72%), Sr (43–55%), Tl (33–43%), Ti (32–41%), U (31–44%), Ho (29–69%), Cr (26–64%), Zr (26–58%), and Ag (25–52%). Dietary intake estimations for wheat flakes have been calculated. A one hundred gram portion of wheat flakes contributed to the RDA or AI values of particular elements for men in the following order: Mn (143%) > Mo > Cu > Mg ≥ Cr > Fe > Se > Zn > P ≥ Ca ≥ K > Na (less than 0.1%); for women: Mn (183%) > Mo > Cu > Cr ≥ Mg > Fe > Zn > Se > P ≥ Ca ≥ K > Na (below 0.1%). For pregnant women aged 19–30, the contributions were as follows: Mn (165%) > Mo > Cu > Mg > Cr > Zn ≥ Se > Fe > P ≥ Ca > K > Na (below 0.1%); for lactating women the contributions were: Mn (127%) > Mo > Cu > Mg > Cr > Fe > Zn ≥ Se > P ≥ Ca > K > Na (below 0.1%). Concerning toxic elements, the highest estimated contribution of wheat flakes to the PTMI value was for Cd and did not exceed 9% for a person weighing 60 kg. The contribution values of all toxic elements were within the limits set by FAO/WHO; therefore, the consumption of an average amount wheat flakes should not pose a health risk and may be recommended as a new component of human staple food. *In vitro* digestibility values for the flakes of between 87.4 and 90.5% were employed to calculate the retention factor for each individual element to assess what proportion of their concentration was still present in the undigested part of the wheat flakes. The highest RF values were found for V (63–92%), Y (57–96%), Ce (43–76%), Pb (34–58%), Tl (32–70%), Ta (31–66%), and Ge (30–49%). The higher RF_max_ value for Ho (62%) should be highlighted as well. The results have shown that K, Mg, P, Zn, Ba, Bi, Ga, Sb, Cu, Ni, and As appeared to be easily released from wheat flake matrices and could be more accessible for absorption by the human tract. It seems that the flaking processes facilitated the release of Zn and Fe from the complex formed with phytic acid in the matrices of the wheat grains. The metal pollution indexes for wheat flakes (0.016–0.024) were established to be lower than wheat grains (0.024–0.033), which may be a consequence of metals leaching during the flaking. This study also emphasizes that 15–25% of the metal pollution index values assessed for native flakes remained in the undigested portion of the flakes that passed further into the large intestine. Providing a considerable consumption of cereals, this finding deserves appropriate attention in future research and practice.

## Figures and Tables

**Table 1:** Minerals and trace elements determined in non-traditional wheat grains and flakes.

Analyte	Native Grains					Native Flakes				
	Samtrot	Tirol	Dickkopf	Rotkorn	Megali	Samtrot	Tirol	Dickkopf	Rotkorn	Megali
(µg/g)										
^23^Na	4.13 ± 0.10 ^a^	4.42 ± 0.10 ^b^	10.6 ± 0.2 ^c^	6.01 ± 0.10 ^d^	4.44 ± 0.12 ^b^	1.33 ± 0.05 ^A^	1.35 ± 0.04 ^A^	5.51 ± 0.20 ^B^	1.74 ± 0.04 ^C^	1.26 ± 0.04 ^D^
^24^Mg	1020 ± 20 ^a^	978 ± 15 ^b^	981 ± 15 ^b^	1090 ± 20 ^c^	878 ± 15 ^d^	658 ± 15 ^A^	690 ± 12 ^B^	758 ± 10 ^C^	967 ± 15 ^D^	679 ± 12 ^E^
^27^Al	1.56 ± 0.03 ^a^	2.05 ± 0.10 ^b^	1.60 ± 0.06 ^a,d^	2.33 ± 0.04 ^c^	1.67 ± 0.05 ^d^	1.45 ± 0.05 ^A^	1.86 ± 0.06 ^B^	1.52 ± 0.03 ^C^	2.01 ± 0.04 ^D^	1.59 ± 0.02 ^E^
^31^P	192 ± 5 ^a^	192 ± 5 ^a^	191 ± 4 ^a^	205 ± 4 ^b^	176 ± 2 ^c^	129 ± 3 ^A^	150 ± 5 ^B^	154 ± 5 ^B^	172 ± 5 ^C^	140 ± 5 ^D^
^32^S	6.11 ± 0.10 ^a^	6.63 ± 0.12 ^b^	6.51 ± 0.12 ^c^	7.52 ± 0.12 ^d^	6.24 ± 0.14 ^e^	4.45 ± 0.20 ^A^	5.12 ± 0.20 ^B^	5.70 ± 0.20 ^C^	6.55 ± 0.12 ^D^	5.17 ± 0.10 ^B^
^39^K	574 ± 10 ^a^	651 ± 12 ^b^	725 ± 20 ^c^	744 ± 14 ^d^	728 ± 12 ^c^	424 ± 10 ^A^	589 ± 15 ^B^	616 ± 14 ^C^	710 ± 12 ^D^	621 ± 12 ^C^
^40^Ca	261 ± 5 ^a^	270 ± 6 ^b^	302 ± 6 ^c^	324 ± 5 ^d^	300 ± 5 ^c^	141 ± 3 ^A^	171 ± 5 ^B^	204 ± 7 ^C^	244 ± 6 ^D^	195 ± 5 ^E^
^48^Ti	2.00 ± 0.10 ^a^	2.78 ± 0.11 ^b^	2.24 ± 0.10 ^c^	3.06 ± 0.20 ^d^	2.26 ± 0.14 ^c^	1.18 ± 0.10 ^A^	1.75 ± 0.07 ^B^	1.52 ± 0.07 ^C^	2.04 ± 0.02 ^D^	1.41 ± 0.02 ^E^
^55^Mn	38.7 ± 0.5 ^a^	31.9 ± 0.5 ^b^	25.2 ± 0.4 ^c^	36.8 ± 0.5 ^d^	31.5 ± 0.5 ^b^	29.2 ± 0.5 ^A^	29.3 ± 0.6 ^A^	20.2 ± 0.3 ^B^	32.9 ± 0.4 ^C^	25.8 ± 0.3 ^D^
^57^Fe	12.3 ± 0.1 ^a^	17.6 ± 0.2 ^b^	11.9 ± 0.2 ^c^	11.2 ± 0.3 ^d^	10.2 ± 0.2 ^e^	7.89 ± 0.15 ^A^	12.5 ± 0.3 ^B^	9.07 ± 0.20 ^C^	9.65 ± 0.10 ^D^	7.46 ± 0.10 ^E^
^63^Cu	8.56 ± 0.05 ^a^	4.57 ± 0.05 ^b^	9.73 ± 0.05 ^c^	6.45 ± 0.10 ^d^	8.16 ± 0.12 ^e^	5.28 ± 0.12 ^A^	3.79 ± 0.12 ^B^	6.25 ± 0.14 ^C^	5.51 ± 0.07 ^D^	5.00 ± 0.05 ^E^
^66^Zn	9.01 ± 0.20 ^a^	7.45 ± 0.20 ^b^	15.4 ± 0.3 ^c^	7.38 ± 0.20 ^b^	7.89 ± 0.10 ^d^	6.37 ± 0.30 ^A,D^	6.07 ± 0.20 ^B^	9.67 ± 0.20 ^C^	6.34 ± 0.10 ^A^	6.44 ± 0.10 ^D^
(ng/g)										
^7^Li	30.8 ± 0.3 ^a^	55.2 ± 0.7 ^b^	59.8 ± 1.0 ^c^	60.8 ± 0.6 ^d^	35.7 ± 0.4 ^e^	16.8 ± 0.4 ^A^	42.1 ± 0.9 ^B^	43.6 ± 1.0 ^C^	30.1 ± 0.3 ^D^	14.7 ± 0.2 ^E^
^9^Be	0.13 ± 0.02 ^a^	0.05 ± 0.01 ^b^	0.07 ± 0.01 ^c^	0.07 ± 0.01 ^c^	0.09 ± 0.01 ^d^	0.11 ± 0.01 ^A^	≤0.04	0.05 ± 0.01 ^B^	≤0.04	0.06 ± 0.01 ^B^
^11^B	242 ± 4 ^a^	231 ± 5 ^b^	282 ± 5 ^c^	393 ± 5 ^d^	279 ± 4 ^c^	224 ± 4 ^A^	217 ± 6 ^B^	234 ± 4 ^C^	341 ± 6 ^D^	260 ± 4 ^E^
^45^Sc	65.6 ± 0.4 ^a^	65.1 ± 0.5 ^a^	73.6 ± 0.5 ^b^	73.7 ± 0.4 ^b^	74.4 ± 0.4 ^c^	51.9 ± 1.0 ^A^	58.9 ± 1.2 ^B^	63.4 ± 1.5 ^C^	67.8 ± 1.2 ^D^	62.6 ± 1.2 ^E^
^51^V	23.5 ± 0.2 ^a^	169 ± 5 ^b^	25.3 ± 0.3 ^c^	65.9 ± 0.7 ^d^	20.5 ± 0.2 ^e^	20.2 ± 0.3 ^A^	112 ± 4 ^B^	21.4 ± 0.2 ^C^	58.3 ± 0.8 ^D^	17.4 ± 0.3 ^E^
^52^Cr	169 ± 2 ^a^	100 ± 4 ^b^	142 ± 5 ^c^	98.6 ± 1.0 ^d^	117 ± 3 ^e^	60.5 ± 0.7 ^A^	74.5 ± 0.7 ^B^	67.0 ± 0.7 ^C^	70.3 ± 0.8 ^D^	61.1 ± 0.7 ^E^
^59^Co	9.51 ± 0.20 ^a^	21.5 ± 0.3 ^b^	8.61 ± 0.40 ^c^	8.75 ± 0.25 ^c^	9.17 ± 0.12 ^d^	7.19 ± 0.50 ^A^	15.5 ± 0.6 ^B^	6.11 ± 0.40 ^C^	7.23 ± 0.20 ^A^	7.39 ± 0.20 ^D^
^60^Ni	299 ± 7 ^a^	169 ± 6 ^b^	286 ± 8 ^c^	291 ± 5 ^c^	115 ± 3 ^d^	197 ± 8 ^A^	138 ± 7 ^B^	183 ± 6 ^C^	208 ± 8 ^D^	62.8 ± 3.4 ^E^
^71^Ga	13.6 ± 0.2 ^a^	16.9 ± 0.3 ^b^	13.4 ± 0.3 ^a^	17.0 ± 0.3 ^b^	12.6 ± 0.2 ^c^	9.25 ± 0.32 ^A^	14.7 ± 0.42 ^B^	11.0 ± 0.5 ^C^	15.0 ± 0.5 ^D^	9.56 ± 0.30 ^E^
^73^Ge	4.95 ± 0.08 ^a^	4.36 ± 0.09 ^b^	4.85 ± 0.09 ^c^	5.99 ± 0.10 ^d^	4.29 ± 0.09 ^e^	4.33 ± 0.20 ^A^	3.87 ± 0.20 ^B^	4.35 ± 0.20 ^A^	5.03 ± 0.03 ^C^	3.90 ± 0.05 ^B^
^75^As	10.7 ± 0.2 ^a^	17.5 ± 0.3 ^b^	8.03 ± 0.10 ^c^	6.70 ± 0.20 ^d^	10.7 ± 0.3 ^a^	9.28 ± 0.10 ^A^	10.9 ± 0.2 ^B^	6.40 ± 0.20 ^C^	5.86 ± 0.14 ^D^	8.86 ± 0.20 ^E^
^77^Se	36.6 ± 0.3 ^a^	29.4 ± 0.5 ^b^	65.3 ± 1.0 ^c^	36.5 ± 0.5 ^a^	25.3 ± 0.4 ^d^	32.8 ± 0.5 ^A^	20.8 ± 0.7 ^B^	56.3 ± 0.5 ^C^	32.8 ± 0.5 ^A^	20.8 ± 0.4 ^B^
^88^Sr	1460 ± 20 ^a^	2500 ± 30 ^b^	1850 ± 30 ^c^	2760 ± 30 ^d^	1650 ± 20 ^e^	756 ± 10 ^A^	1130 ± 20 ^B^	1060 ± 30 ^C^	1430 ± 30 ^D^	809 ± 15 ^E^
^89^Y	0.53 ± 0.02 ^a^	0.55 ± 0.03 ^b^	0.49 ± 0.04 ^c^	1.08 ± 0.02 ^d^	0.41 ± 0.02 ^e^	0.42 ± 0.05 ^A,C^	0.38 ± 0.03 ^B^	0.40 ± 0.04 ^C^	0.72 ± 0.04 ^D^	0.35 ± 0.02 ^E^
^90^Zr	42.7 ± 0.5 ^a^	21.0 ± 0.4 ^b^	45.0 ± 0.6 ^c^	12.1 ± 0.3 ^d^	35.1 ± 0.6 ^e^	19.7 ± 0.5 ^A^	12.3 ± 0.4 ^B^	33.1 ± 0.4 ^C^	6.14 ± 0.06 ^D^	14.8 ± 0.2 ^E^
^95^Mo	568 ± 5 ^a^	460 ± 5 ^b^	657 ± 5 ^c^	671 ± 10 ^d^	326 ± 8 ^e^	458 ± 7 ^A^	409 ± 8 ^B^	574 ± 8 ^C^	614 ± 11 ^D^	282 ± 7 ^E^
^107^Ag	4.40 ± 0.20 ^a^	1.25 ± 0.10 ^b^	6.70 ± 0.25 ^c^	4.78 ± 0.15 ^d^	7.84 ± 0.22 ^e^	2.12 ± 0.10 ^A^	0.89 ± 0.04 ^B^	3.98 ± 0.12 ^C^	3.60 ± 0.10 ^D^	5.07 ± 0.12 ^E^
^111^Cd	19.7 ± 0.3 ^a^	9.35 ± 0.20 ^b^	52.9 ± 1.0 ^c^	28.4 ± 0.7 ^d^	6.66 ± 0.3 ^e^	15.4 ± 0.9 ^A^	7.84 ± 0.09 ^B^	45.2 ± 1.0 ^C^	26.1 ± 0.9 ^D^	5.60 ± 0.5 ^E^
^118^Sn	146 ± 4 ^a^	314 ± 7 ^b^	143 ± 3 ^a^	530 ± 7 ^c^	180 ± 2 ^d^	107 ± 3 ^A^	178 ± 6 ^B^	110 ± 6 ^A^	467 ± 10 ^C^	117 ± 4 ^D^
^121^Sb	4.66 ± 0.22 ^a^	4.15 ± 0.22 ^b^	4.76 ± 0.22 ^a^	4.28 ± 0.12 ^c^	6.14 ± 0.14 ^d^	4.15 ± 0.10 ^A^	3.61 ± 0.20 ^B^	4.03 ± 0.20 ^C^	3.21 ± 0.10 ^D^	5.45 ± 0.12 ^E^
^133^Cs	0.31 ± 0.05 ^a^	1.65 ± 0.07 ^b^	0.36 ± 0.05 ^c^	0.94 ± 0.05 ^d^	0.36 ± 0.04 ^c^	0.28 ± 0.03 ^A,E^	1.35 ± 0.03 ^B^	0.31 ± 0.04 ^C,E^	0.78 ± 0.04 ^D^	0.29 ± 0.03 ^E^
^137^Ba	283 ± 4 ^a^	254 ± 5 ^b^	978 ± 11 ^c^	1140 ± 20 ^d^	274 ± 5 ^e^	218 ± 7 ^A^	240 ± 7 ^B^	810 ± 12 ^C^	1050 ± 15 ^D^	228 ± 7 ^E^
^140^Ce	1.89 ± 0.05 ^a^	22.2 ± 0.2 ^b^	1.19 ± 0.05 ^c^	5.68 ± 0.10 ^d^	1.54 ± 0.04 ^e^	0.63 ± 0.03 ^A^	6.88 ± 0.30 ^B^	0.63 ± 0.05 ^A^	1.58 ± 0.10 ^C^	0.60 ± 0.04 ^A^
^159^Tb	≤0.03	0.38 ± 0.02 ^a^	≤0.04	0.05 ± 0.01 ^b^	≤0.03	≤0.02	0.21 ± 0.02	≤0.03	≤0.04	≤0.02
^165^Ho	≤0.03	0.29 ± 0.02 ^a^	≤0.03	0.07 ± 0.01 ^b^	≤0.03	≤0.02	0.09 ± 0.01 ^A^	≤0.02	0.05 ± 0.01 ^B^	≤0.02
^181^Ta	9.32 ± 0.12 ^a^	12.9 ± 0.2 ^b^	9.65 ± 0.13 ^c^	7.36 ± 0.09 ^d^	8.07 ± 0.07 ^e^	8.26 ± 0.13 ^A^	9.83 ± 0.14 ^B^	8.72 ± 0.12 ^C^	6.10 ± 0.20 ^D^	7.50 ± 0.20 ^E^
^202^Hg	20.9 ± 0.3 ^a^	15.5 ± 0.4 ^b^	20.6 ± 0.4 ^a^	14.0 ± 0.3 ^c^	19.9 ± 0.5 ^d^	14.8 ± 0.5 ^A^	11.3 ± 0.3 ^B^	12.8 ± 0.4 ^C^	12.2 ± 0.2 ^D^	12.4 ± 0.2 ^D^
^205^Tl	0.07 ± 0.01 ^a,c^	0.08 ± 0.02 ^a,b,c^	0.09 ± 0.01 ^b^	0.07 ± 0.01 ^a,c^	0.06 ± 0.01 ^c^	≤0.04	0.05 ± 0.05 ^A^	0.06 ± 0.01 ^A^	≤0.04	≤0.04
^208^Pb	68.3 ± 1.0 ^a^	72.1 ± 1.2 ^b^	50.2 ± 1.4 ^c^	61.0 ± 1.0 ^d^	40.8 ± 1.4 ^e^	57.8 ± 1.4 ^A^	46.1 ± 1.1 ^B^	44.9 ± 1.1 ^C^	34.4 ± 1.0 ^D^	35.9 ± 1.0 ^E^
^209^Bi	2.84 ± 0.12 ^a^	2.30 ± 0.05 ^b^	1.24 ± 0.02 ^c^	2.01 ± 0.04 ^d^	1.38 ± 0.02 ^e^	1.44 ± 0.04 ^A^	1.76 ± 0.05 ^B^	0.73 ± 0.02 ^C^	1.41 ± 0.05 ^A^	0.79 ± 0.02 ^D^
^238^U	0.65 ± 0.02 ^a^	0.21 ± 0.05 ^b^	0.52 ± 0.03 ^c^	0.75 ± 0.05 ^d^	1.03 ± 0.03 ^e^	0.45 ± 0.02 ^A^	0.13 ± 0.01 ^B^	0.35 ± 0.03 ^C^	0.49 ± 0.03 ^D^	0.58 ± 0.04 ^E^

All results are presented on a dry matter basis as means ± SD, n = 5 (the mean of five measurements). Means within a line with at least one identical lowercase superscript letter (raw grains) do not differ significantly (*p* ≥ 0.05); means within a line with at least one identical capitalized superscript letter (wheat flakes) do not differ significantly (*p* ≥ 0.05).

**Table 2:** Minerals and trace elements determined in undigested parts of non-traditional wheat flakes.

	Undigested Parts of Flakes				
Analyte	Samtrot	Tirol	Dickkopf	Rotkorn	Megali
(µg/g)					
^23^Na	3.41 ± 0.15 ^a^	3.75 ± 0.20 ^b^	25.5 ± 0.3 ^c^	3.57 ± 0.12 ^d^	2.90 ± 0.10 ^e^
^24^Mg	117 ± 3 ^a^	252 ± 7 ^b^	102 ± 3 ^c^	311 ± 6 ^d^	112 ± 2 ^e^
^27^Al	4.02 ± 0.12 ^a^	4.90 ± 0.12 ^b^	2.31 ± 0.10 ^c^	6.23 ± 0.20 ^d^	2.10 ± 0.05 ^e^
^31^P	38.9 ± 0.3 ^a^	63.1 ± 0.5 ^b^	30.4 ± 0.4 ^c^	65.7 ± 0.7 ^d^	32.8 ± 0.5 ^e^
^32^S	6.78 ± 0.30 ^a^	16.8 ± 0.5 ^b^	7.02 ± 0.20 ^c^	22.8 ± 0.4 ^d^	6.41 ± 0.20 ^e^
^39^K	65.6 ± 0.6 ^a^	77.4 ± 0.6 ^b^	63.2 ± 0.6 ^c^	71.5 ± 0.7 ^d^	64.5 ± 0.8 ^e^
^40^Ca	253 ± 5 ^a^	490 ± 8 ^b^	269 ± 6 ^c^	505 ± 10 ^d^	250 ± 5 ^a^
^48^Ti	2.46 ± 0.09 ^a^	2.66 ± 0.10 ^b^	2.40 ± 0.11 ^a,d^	2.94 ± 0.04 ^c^	2.38 ± 0.02 ^d^
^55^Mn	103 ± 4 ^a^	14.3 ± 0.4 ^b^	50.9 ± 0.5 ^c^	23.5 ± 0.2 ^d^	75.2 ± 0.8 ^e^
^57^Fe	12.1 ± 0.2 ^a^	14.7 ± 0.3 ^b^	11.2 ± 0.3 ^c^	19.4 ± 0.3 ^d^	11.7 ± 0.2 ^e^
^63^Cu	3.76 ± 0.10 ^a^	3.72 ± 0.10 ^a^	3.71 ± 0.12 ^a^	5.95 ± 0.20 ^b^	3.39 ± 0.12 ^c^
^66^Zn	2.46 ± 0.10 ^a^	3.86 ± 0.12 ^b^	2.55 ± 0.10 ^c^	5.52 ± 0.22 ^d^	3.73 ± 0.10 ^e^
(ng/g)					
^7^Li	15.8 ± 0.5 ^a^	58.1 ± 1.2 ^b^	19.3 ± 0.4 ^c^	61.3 ± 0.6 ^d^	18.3 ± 0.3 ^e^
^9^Be	0.11 ± 0.02 ^a^	0.15 ± 0.02 ^b,e^	0.13 ± 0.02 ^c^	0.17 ± 0.02 ^d,e^	0.16 ± 0.02 ^e^
^11^B	202 ± 5 ^a^	455 ± 10 ^b^	162 ± 7 ^c^	369 ± 10 ^d^	136 ± 4 ^e^
^45^Sc	65.5 ± 1.0 ^a^	160 ± 5 ^b^	60.1 ± 1.4 ^c^	155 ± 3 ^d^	64.3 ± 1.2 ^e^
^51^V	162 ± 4 ^a^	593 ± 10 ^b^	144 ± 5 ^c^	327 ± 6 ^d^	142 ± 3 ^c^
^52^Cr	136 ± 5 ^a^	259 ± 7 ^b^	121 ± 5 ^c^	267 ± 7 ^d^	90.7 ± 1.5 ^e^
^59^Co	26.9 ± 0.7 ^a^	33.6 ± 0.9 ^b^	19.5 ± 0.6 ^c^	30.6 ± 0.8 ^d^	19.1 ± 0.4 ^e^
^60^Ni	253 ± 8 ^a^	123 ± 5 ^b^	153 ± 6 ^c^	115 ± 5 ^d^	130 ± 5 ^e^
^71^Ga	3.31 ± 0.10 ^a^	3.75 ± 0.20 ^b^	3.33 ± 0.30 ^a^	4.82 ± 0.16 ^c^	3.08 ± 0.12 ^d^
^73^Ge	13.6 ± 0.3 ^a^	11.9 ± 0.2 ^b^	13.6 ± 0.2 ^a^	21.8 ± 0.5 ^c^	10.5 ± 0.2 ^d^
^75^As	8.37 ± 0.12 ^a^	14.2 ± 0.2 ^b^	7.77 ± 0.15 ^c^	14.7 ± 0.4 ^d^	6.46 ± 0.10 ^e^
^77^Se	54.0 ± 0.6 ^a^	94.1 ± 1.0 ^b^	45.6 ± 0.6 ^c^	54.1 ± 0.6 ^d^	44.6 ± 0.6 ^e^
^88^Sr	1050 ± 20 ^a^	1300 ± 30 ^b^	881 ± 12 ^c^	1370 ± 20 ^d^	728 ± 10 ^e^
^89^Y	3.56 ± 0.25 ^a^	1.71 ± 0.10 ^b^	4.04 ± 0.10 ^c^	3.15 ± 0.10 ^d^	2.53 ± 0.10 ^e^
^90^Zr	35.1 ± 0.6 ^a^	29.2 ± 0.6 ^b^	26.3 ± 0.6 ^c^	4.24 ± 0.04 ^d^	26.8 ± 0.2 ^e^
^95^Mo	655 ± 10 ^a^	287 ± 4 ^b^	604 ± 10 ^c^	490 ± 8 ^d^	383 ± 8 ^e^
^107^Ag	6.45 ± 0.17 ^a^	1.72 ± 0.10 ^b^	18.5 ± 0.4 ^c^	17.0 ± 0.3 ^d^	20.1 ± 0.3 ^e^
^111^Cd	18.7 ± 0.4 ^a^	21.5 ± 0.3 ^b^	123 ± 6 ^c^	74.2 ± 1.2 ^d^	13.3 ± 0.2 ^e^
^118^Sn	281 ± 2 ^a^	412 ± 5 ^b^	504 ± 9 ^c^	1150 ± 30 ^d^	413 ± 8 ^b^
^121^Sb	3.21 ± 0.30 ^a^	2.18 ± 0.25 ^b^	3.27 ± 0.30 ^a^	2.94 ± 0.04 ^c^	2.84 ± 0.04 ^d^
^133^Cs	0.41 ± 0.02 ^a^	0.72 ± 0.04 ^b^	0.39 ± 0.05 ^c^	0.81 ± 0.03 ^d^	0.36 ± 0.02 ^e^
^137^Ba	55.2 ± 1.5 ^a^	177 ± 7 ^b^	43.0 ± 0.8 ^c^	163 ± 7 ^d^	53.5 ± 1.5 ^e^
^140^Ce	4.00 ± 0.10 ^a^	26.3 ± 0.5 ^b^	4.18 ± 0.14 ^c^	6.12 ± 0.12 ^d^	4.01 ± 0.06 ^a^
^159^Tb	0.06 ± 0.01 ^a^	0.17 ± 0.02 ^b^	0.06 ± 0.01 ^a^	0.08 ± 0.01 ^c^	0.05 ± 0.01 ^a^
^165^Ho	0.11 ± 0.01 ^a^	9.83 ± 0.22 ^b^	0.13 ± 0.02 ^c^	0.11 ± 0.01 ^a^	0.07 ± 0.01 ^d^
^181^Ta	23.7 ± 0.5 ^a^	45.2 ± 0.7 ^b^	37.1 ± 0.7 ^c^	35.9 ± 0.7 ^d^	25.4 ± 0.6 ^e^
^202^Hg	58.6 ± 0.5 ^a^	30.8 ± 0.3 ^b^	26.8 ± 0.3 ^c^	41.5 ± 0.5 ^d^	24.3 ± 0.2 ^e^
^205^Tl	0.22 ± 0.02 ^a^	0.20 ± 0.01 ^b^	0.20 ± 0.02 ^b^	0.25 ± 0.01 ^c^	0.22 ± 0.01 ^a^
^208^Pb	215 ± 4 ^a^	126 ± 2 ^b^	225 ± 5 ^c^	179 ± 5 ^d^	185 ± 5 ^e^
^209^Bi	1.61 ± 0.03 ^a^	0.33 ± 0.02 ^b^	0.28 ± 0.02 ^c^	0.53 ± 0.03 ^d^	0.13 ± 0.01 ^e^
^238^U	2.02 ± 0.10 ^a^	0.23 ± 0.02 ^b^	1.12 ± 0.12 ^c^	1.08 ± 0.04 ^c^	2.15 ± 0.05 ^d^

All results are presented in dry matter as means ± SD, n = 5 (the mean of five measurements). Means within a line with at least one identical lowercase superscript letter do not differ significantly (*p* ≥ 0.05).

## Data Availability

Data are contained within the article.

## Supplementary Materials

The following supporting information can be downloaded at: https://www.mdpi.com/article/10.3390/nu15112509/s1, Table S1: The resulting values for certified materials measured by using ICP-MS; Table S2: RDA, AI*, PTWI, and PTMI* reference values for individual life stage groups defined by the IOM and the JECFA; Table S3: Dry matter and ash contents and dry matter digestibility (DMD) values for non-traditional wheat flakes; Table S4: Daily intakes for essential nutrients estimated from 100 g of non-traditional wheat flakes (women aged 19–30 and pregnant and lactating women aged 19–30); Table S5: Daily intakes for essential nutrients estimated from 100 g of non-traditional wheat flakes (women aged 31–50, 51–70, and ≥70); Table S6: Daily intake for essential nutrients estimated from 100 g of non-traditional wheat flakes (men aged 19–30, 31–50, 51–70, and ≥70); Table S7: Maximum dietary intake estimations for non-traditional wheat flakes in relation to RDA (AI*) and PTWI (PTMI*) values for essential and toxic elements; Table S8: Dietary intake estimations for toxic elements (based on bw); Table S9: Daily intakes for non-essential and toxic elements; Table S10: Metal pollution index (MPI) for non-traditional wheat grains, flakes, the undigested parts of flakes, and the remaining parts of the metal pollution index (RP_MPI_).

## References

[1] Drawbridge P.C., Apea-Bah F., Hornung P.S., Beta T. (2021). Bioaccessibility of phenolic acids in Canadian hulless barley varieties. Food Chem..

[2] Lemmens E., Deleu L.J., De Brier N., Smolders E., Delcour J.A. (2021). Mineral bio-accessibility and intrinsic saccharides in breakfast flakes manufactured from sprouted wheat. LWT—Food Sci. Technol..

[3] Kiewlitz J., Rybicka I. (2020). Minerals and their bioavailability in relation to dietary fiber, phytates and tannins from gluten and gluten-free flakes. Food Chem..

[4] Liu Z., Wang H., Wang X.-E., Xu H., Gao D., Zhang G., Chen P., Liu D. (2008). Effect of wheat pearling on flour phytate activity, phytic acid, iron and zinc content. LWT—Food Sci. Technol..

[5] Ficco D.B.M., Borrelli G.M., Miedico O., Giovanniello V., Tarallo M., Pompa C., De Vita P., Chiaravalle A.E. (2020). Effect of grain debranning on bioactive compounds, antioxidant capacity and essential and toxic trace elements in purple durum wheats. LWT—Food Sci. Technol..

[6] Do Prado Ferreira M., Teixeira Tarley C.R. (2021). Bioaccessibility estimation of metallic macro and micronutrients Ca, Mg, Zn, Fe, Cu and Mn in flours of oat and passion fruit peel. LWT—Food Sci. Technol..

[7] Dost K., Tokul O. (2006). Determination of phytic acid in wheat and wheat products by reverse phase high performance liquid chromatography. Anal. Chim. Acta.

[8] Affonfere M., Chadare F.J., Fassinou F.T.K., Linnemann A.R., Duodu K.G. (2023). *In-vitro* digestibility methods and factors affecting minerals bioavailability: A review. Food Rev. Int..

[9] Adetola O.Y., Kruger J., White Z., Taylor J.R.N. (2019). Comparison between food-to-food fortification of pearl millet porridge with moringa leaves and baobab fruit and with adding ascorbic and citric acid on iron, zinc and other mineral bioaccessibility. LWT—Food Sci. Technol..

[10] Brouns F. (2022). Phytic acid and whole grains for health controversy. Nutrients.

[11] Sęczyk Ł., Sugier D., Świeca M., Gawlik-Dziki U. (2021). The effect of *in vitro* digestion, food matrix, and hydrothermal treatment on the potential bioaccessibility of selected phenolic compounds. Food Chem..

[12] Liu K., Zheng J., Wang X., Chen F. (2019). Effect of household cooking process on mineral, vitamin B, and phytic acid contents and mineral bioaccessibility in rice. Food Chem..

[13] Koláčková T., Sumczynski D., Minařík A., Yalçin E., Orsavová J. (2022). The effect of *in vitro* digestion on matcha tea *(Camellia sinensis)* active components and antioxidant activity. Antioxidants.

[14] Sumczynski D., Koubová E., Sneyd J., Erb-Weber S., Orsavová J. (2018). Preparation of non-traditional Dickkopf and Richard wheat flakes: Phenolic and vitamin profiles and antioxidant activity. LWT—Food Sci. Technol..

[15] AOAC (2007). Association of Official Analytical Chemists International.

[16] Sumczynski D., Koubová E., Šenkárová L., Orsavová J. (2018). Rice flakes produced from commercial wild rice: Chemical composition, vitamin B compounds, mineral and trace element contents and their dietary intake evaluation. Food Chem..

[17] Institute of Medicine (1997). Dietary Reference Intakes for Calcium, Phosphorus, Magnesium, Vitamin D, and Fluoride.

[18] Institute of Medicine (2000). Dietary Reference Intakes for Vitamin C, Vitamin E, Selenium, and Carotenoids.

[19] Institute of Medicine (2001). Dietary Reference Intakes for Vitamin A, Vitamin K, Arsenic, Boron, Chromium, Copper, Iodine, Iron, Manganese, Molybdenum, Nickel, Silicon, Vanadium, and Zinc.

[20] Institute of Medicine (2005). Dietary Reference Intakes for Water, Potassium, Sodium, Chloride, and Sulfate.

[21] Joint FAO/WHO Expert Committee on Food Additives (2006). Evaluation of Certain Contaminants: Sixty-Fourth Report of the Joint FAO/WHO Expert Committee on Food Additives.

[22] Joint FAO/WHO Expert Committee on Food Additives (2011). Evaluation of Certain Contaminants in Food: Seventy-Second Report of the Joint FAO/WHO Expert Committee on Food Additives.

[23] Joint FAO/WHO Expert Committee on Food Additives (2011). Evaluation of Certain Food Additives and Contaminants: Seventy-Fourth Report of the Joint FAO/WHO Expert Committee on Food Additives.

[24] Joint FAO/WHO Expert Committee on Food Additives (2013). Evaluation of Certain Food Additives and Contaminants: Seventy-Seventh Report of the Joint FAO/WHO Expert Committee on Food Additives.

[25] Orecchio S., Amorello D., Raso M., Barreca S., Lino C., Di Gaudio F. (2014). Determination of trace elements in gluten-free food for celiac people by ICP-MS. Microchem. J..

[26] Singh P.K., Yadav J.S., Kumar I., Kumar U., Sharma R.K. (2022). Carpet industry irrigational sources risk assessment: Heavy metal contaminated vegetables and cereal crops in northern India. Toxicol. Rep..

[27] (2020). Regulation for Cereal and Cereal Products, Pasta and Bakery Products.

[28] Sumczynski D., Bubelova Z., Sneyd J., Erb-Weber S., Mlcek J. (2015). Total phenolics, flavonoids, antioxidant activity, crude fibre and digestibility in non-traditional wheat flakes and muesli. Food Chem..

[29] Qiao F.-Q., Wang F., Ren L.-P., Zhou Z.-M., Meng Q.-X., Bao Y.-H. (2015). Effect of steam-flaking on chemical compositions, starch gelatinization, *in vitro* fermentability, and energetic values of maize, wheat and rice. J. Integr. Aric..

[30] Škrbić B., Čupić S. (2005). Toxic and essential elements in soft wheat grain cultivated in Serbia. Eur. Food Res. Technol..

[31] Karami M., Afyuni M., Khoshgoftarmanesh A.H., Papritz A., Schulin R. (2009). Grain zinc, iron, and copper concentrations of wheat grown in central Iran and their relationships with soil and climate variables. J. Agric. Food Chem..

[32] Bermudez G.M.A., Jasan R., Plá R., Pignata M.L. (2011). Heavy metal and trace element concentrations in wheat grains: Assessment of potential non-carcinogenic health hazard through their consumption. J. Hazard. Mater..

[33] Zvěřina O., Kuta J., Coufalík P., Kosečková P., Komárek J. (2019). Simultaneous determination of cadmium and iron in different kinds of cereal flakes using high-resolution continuum source atomic absorption spectrometry. Food Chem..

[34] (2008). Commission Regulation (EC) No 629/2008 of 2 July 2008 Amending Regulation (EC) No 1881/2006 Maximum Levels for Certain Contaminants in Foodstuffs. https://eur-lex.europa.eu/legal-content/EN/TXT/PDF/?uri=CELEX:32008R0629&qid=1673430422268&from=CS.

[35] (2021). Commission Regulation (EU) 2021/1323 of 10 August 2021 Amending Regulation (EC) No 1881/2006 as Regards Maximum Levels of Cadmium in Certain Foodstuffs. https://eur-lex.europa.eu/legal-content/EN/TXT/PDF/?uri=CELEX:32021R1323&from=EN.

[36] European Food Safety Authority (2012). Cadmium dietary exposure in the European population. EFSA J..

[37] Commission Regulation (EU) No 420/2011 of 29 April 2011 Amending Regulation (EC) No 1881/2006 Setting Maximum Levels for Certain Contaminants in Foodstuffs. https://eur-lex.europa.eu/legal-content/EN/TXT/PDF/?uri=CELEX:32011R0420&qid=1673429826655&from=CS.

[38] Commission Regulation (EU) 2021/1317 of 9 August 2021 Amending Regulation (EC) No 1881/2006 as Regards Maximum Levels of Lead in Certain Foodstuffs. https://eur-lex.europa.eu/legal-content/EN/TXT/PDF/?uri=CELEX:32021R1317&qid=1678878875525&from=CS.

[39] Commission Regulation (EU) 2015/1006 of 25 June 2015 Amending Regulation (EC) No 1881/2006 as Regards Maximum Levels of Inorganic Arsenic in Foodstuffs. https://eur-lex.europa.eu/legal-content/EN/TXT/PDF/?uri=CELEX:32015R1006&qid=1678879433609&from=CS.

[40] Wu G., Ashton J., Simic A., Fang Z., Johnson S.K. (2018). Mineral availability is modified by tannin and phytate content in sorghum flaked breakfast cereals. Food Res. Int..

[41] Kabata-Pendias A. (2011). Trace Elements in Soils and Plants.

[42] Regulation (EU) No 1169/2011 of the European Parliament and of the Council of 25 October 2011 on the Provision of Food Information to Consumers, Amending Regulation (EC) No 1924/2006 and (EC) No 1925/2006 of the European Parliament and of the Council, and Repealing Commission Directive 87/250/EEC, Council Directive 90/496,EEC, Commission Directive 1990/10/EC, Directive 2000/13/EC of the European Parliament and of the Council, Commission Directives 2002/67/EC and 2008/5/EC and Commission Regulation (EC) No 608/2004. https://eur-lex.europa.eu/legal-content/EN/TXT/PDF/?uri=CELEX:02011R1169-20180101&qid=1678014199899&from=CS.

[43] Antoine J.M.R., Hoo Fung L.A., Grant C.N., Dennis H.T., Lalor G.C. (2012). Dietary intake of minerals and trace elements in rice on the Jamaican market. J. Food Compos. Anal..

[44] European Food Safety Authority (2014). European Food Safety Authority. EFSA J..

[45] European Food Safety Authority (2023). Scientific Opinion on the tolerable upper intake level for selenium. EFSA J..

[46] European Food Safety Authority (2015). Scientific Opinion on dietary reference values for copper. EFSA J..

[47] Mathebula M.W., Mandiwana K., Panichev N. (2017). Speciation of chromium in bread and breakfast cereals. Food Chem..

[48] European Food Safety Authority (2014). Scientific Opinion on the risk to public health related to the presence of chromium in food and drinking water. EFSA J..

[49] U.S. Environmental Protection Agency (2022). IRIS Toxicological Review of Hexavalent Chromium (External Review Draft): Support of Summary Information on the Integrated Risk Information System (IRIS).

[50] Dong J.Y., Xun P., He K., Qin L.Q. (2011). Magnesium intake and risk of type 2 diabetes: Meta-analysis of prospective cohort studies. Diabetes Care.

[51] Kumar N. (2007). Nutritional neuropathies. Neurol. Clin..

[52] European Food Safety Authority (2023). Re-evaluation of the existing health-based guidance values for copper and exposure assessment from all sources. EFSA J..

[53] European Food Safety Authority (2013). Scientific Opinion on dietary reference values for molybdenum. EFSA J..

[54] (2013). Deutsche Gesellschaft für Ernährung—Österreichische Gesellschaft für Ernährung, Schweizerische Gesellschaft für Ernährungsforschung, Schdweizerische Vereinigung für Ernährung: Referenzwerte für die Nährstoffzufuhr.

[55] European Food Safety Authority (2013). Scientific Opinion on dietary reference values for manganese. EFSA J..

[56] More S.J., Bampidis V., Benford D., Bragard C., Halldorsson T.I., Hernández-Jerez A.F., Bennekou S.H., Koutsoumanis K., Lambré C., EFSA Scientific Committee (2020). Content of toxic elements in 12 group of rice products available on Polish market: Human health risk assessment. Foods.

[57] Agency for Toxic Substances and Disease Registry (2022). ATSDR’s Substance Priority List. https://www.atsdr.cdc.gov/spl/index.html#2022spl.

[58] European Food Safety Authority (2009). Cadmium in food. Scientific Opinion of the panel on contaminants in the food chain. EFSA J..

[59] European Food Safety Authority (2011). Scientific Opinion. Statement on tolerable weekly intake for cadmium. EFSA J.

[60] European Food Safety Authority (2012). Scientific opinion on the risk for public health related to the presence of mercury and methylmercury in food. EFSA J..

[61] European Food Safety Authority (2008). Safety of aluminium from dietary intake. Scientific Opinion of the Panel on Food Additives, Flavourings, Processing Aids and Food Contact Materials (AFC). EFSA J..

[62] Filippini T., Tancredi S., Malagoli C., Cilloni S., Malavolti M., Violi F., Vescovi L., Bargellini A., Vinceti M. (2019). Aluminium and tin: Food contamination and dietary intake in an Italian population. J. Trace. Elem Med. Biol..

[63] Koubová E., Sumczynski D., Šenkárová L., Orsavová J., Fišera M. (2018). Dietary intakes of minerals, essential and toxic trace elements for adults from *Eragrostis tef* L.: A nutritional assessment. Nutrients.

[64] European Food Safety Authority (2005). Opinion of the Scientific Panel on Dietetic Products, Nutrition and Allergies on a request from the Commission related to the tolerable upper intake level of tin. EFSA J..

[65] Commission Regulation (EC) No 1881/2006 of 19 December 2006 Setting Maximum Levels for Certain Contaminants in Foodstuffs. https://eur-lex.europa.eu/legal-content/EN/TXT/PDF/?uri=CELEX:02006R1881-20230101&qid=1678820563695&from=CS.

[66] Joint FAO/WHO Expert Committee on Food Additives (2011). Evaluation of Certain Food Additives and Contaminants: Seventy-Third Report of the Joint FAO/WHO Expert Committee on Food Additives.

[67] European Food Safety Authority (2010). Scientific Opinion on lead in food. EFSA J..

[68] European Food Safety Authority (2012). Scientific Report of EFSA. Lead dietary exposure in the European population. EFSA J..

[69] European Food Safety Authority (2014). Scientific Report of EFSA. Dietary exposure to inorganic arsenic in the European population. EFSA J..

[70] European Food Safety Authority (2009). Scientific Opinion on arsenic in food. EFSA J..

[71] European Food Safety Authority (2015). Scientific Opinion on the risks to public health related to the presence of nickel in food and drinking water. EFSA J..

[72] European Food Safety Authority (2020). Update of the risk assessment of nickel in food and drinking water. EFSA J..

[73] Akinyele I.O., Shokunbi O.S. (2015). Concentrations of Mn, Fe, Cu, Zn, Cr, Cd, Pb, Ni in selected Nigerian tubers, legumes and cereals and estimates of the adult daily intakes. Food Chem..

[74] Drake P.L., Hazelwood K.J. (2005). Exposure-related health effects of silver and silver compounds: A review. Ann. Occup. Hyg..

[75] World Health Organization (2021). Silver in Drinking-Water: Background Document for Development of WHO Guidelines for Drinking-Water Quality.

[76] Dawson P.A., Elliot A., Bowling F.G. (2015). Sulphate in pregnancy. Nutrients.

[77] Kowalczyk E., Givelet L., Amlund H., Sloth J.J., Hansen M. (2022). Risk assessment of rare earth elements, antimony, barium, boron, lithium, tellurium, thallium and vanadium in teas. EFSA J..

[78] World Health Organization (2003). Antimony in Drinking-Water: Background Document for Development of WHO Guidelines for Drinking-Water Quality.

[79] Wu F., Fu Z., Liu B., Mo C., Chen B., Corns W., Liao H. (2011). Health risk associated with dietary co-exposure to high levels of antimony and arsenic in the world’s largest antimony mine area. Sci. Total Environ..

[80] Scientific Committee on Health and Environmental Risks SCHER (2012). Assessment of the Tolerable Daily Intake of Barium.

[81] World Health Organization (2010). Strontium and Strontium Compounds: Concise International Chemical Assessment Document 77.

[82] Kołodziejska B., Stępień J., Kolmas J. (2021). The influence of strontium on bone metabolism and its application in osteoporosis treatment. Int. J. Mol. Sci..

[83] U.S. Environmental Protection Agency (2006). Inorganic Contaminant Accumulation in Potable Water Distribution Systems.

[84] European Food Safety Authority (2006). Tolerable Upper Intake Levels for Vitamins and Minerals: Scientific Committee on Food.

[85] Szklarska D., Rzymski P. (2019). Is lithium a micronutrient? From biological activity and epidemiological observation to food fortification. Biol. Trace Elem. Res..

[86] Schrauzer G.N. (2002). Lithium: Occurrence, dietary intakes, nutritional essentiality. J. Am. Coll. Nutr..

[87] European Food Safety Authority (2004). Opinion of the Scientific Panel on Dietetic Products, Nutrition and Allergies on a request from the Commission related to the tolerable upper intake level of boron (sodium borate and boric acid). EFSA J..

[88] Nielsen F.H. (2014). Update on human health effects of boron. J. Trace Elem. Med. Biol..

[89] National Institutes of Health Boron. Fact Sheet for Health Professionals. https://ods.od.nih.gov/factsheets/Boron-HealthProfessional/.

[90] World Health Organization (1996). Boron: Trace Elements in Human Nutrition and Health.

[91] World Health Organization & International Programme on Chemical Safety (1998). Boron: Environmental Health Criteria 204.

[92] U.S. Environmental Protection Agency (2009). Toxicological Review of Thallium and Compounds.

[93] Cvjetko P., Cvjetko I., Pavlica M. (2010). Thallium toxicity in humans. Arh. Hig. Rada. Toksikol..

[94] European Food Safety Authority (2020). Review of potentially toxic rare earth elements, thallium and tellurium in plant-based foods. EFSA J..

[95] Minekus M., Alminger M., Alvito P., Ballance S., Bohn TO RS TE N., Bourlieu C., Brodkorb A. (2014). Standardised static *in vitro* digestion method suitable for food–an international consensus. Food Funct..

[96] Sharafi K., Nodehi R.N., Mahvi A.H., Pirsaheb M., Nazmara S., Mahmoudi B., Yunesian M. (2019). Bioacessibility analysis of toxic metals in consumed rice through an *in vitro* human digestion model—Comparison of calculated human health risk from raw, cooked and digested rice. Food Chem..

[97] Cioca A.-A., Langerholc T., Tušar L. (2022). Implementation of food matrix effects into chemical food contaminant risk assessment. EFSA J..

[98] Kumar D., Prijanka, Shukla V., Kumar S., Ram R.B., Kumar N. (2020). Metal pollution index and daily dietary intake of metals through consumption of vegetables. Int. J. Environ. Sci. Technol..

[99] Sanaei F., Amin M.M., Alavijeh Z.P., Esfahani R.A., Sadeghi M., Bandarrig N.S., Rezakazemi M. (2021). Health risk assessment of potentially toxic elements intake via food crops consumption: Monte Carlo simulation-based probabilistic and heavy metal pollution index. Environ. Sci. Pollut. Res..

