# Supplementary material for: The Effect of Flake Production and In Vitro Digestion on Releasing Minerals and Trace Elements from Wheat Flakes: The Extended Study of Dietary Intakes for Individual Life Stage Groups
Source: Nutrients. 2023 May 28;15(11):2509. doi: 10.3390/nu15112509 (PMC10255177; doi:10.3390/nu15112509)
Supplement: Supplementary file 1 [file nutrients-15-02509-s001.zip › nutrients-2393874-supplementary.pdf]

Supplementary material

The Effect of Flake Production and *in vitro* Digestion on Releasing Minerals and Trace Elements from Wheat flakes: The Extended Study of Dietary Intakes for Individual Life Stage Group

Table S1: The resulting values of certified materials measured by using ICP-MS.

Reference materials	Elements	Analysed values[a]	Reference values[a,b]	Recovery (%)
Metranal® Green algae	As	38.4±0.5	41±3	94.0
	Ca	1370±20	1380±80	99.0
	Cd	0.022±0.004	0.023±0.004	95.6
	Co	18.2±1.2	18.0±1.6	101.0
	Cu	33.3±1.0	34.0±1.6	97.9
	Fe	283±10	290±20	97.6
	Hg	0.018±0.002	0.017±0.010	105.9
	Mg	1600±20	1580±12	101.3
	Mn	42.6±1.2	43.0±3.4	99.1
	Ni	0.82±0.10	0.80±0.10	102.5
	Pb	0.22±0.02	0.21±0.01	104.8
	Zn	38.1±1.0	38.0±3.0	100.2
NIST 568b Rice flour	Al	4.25 ± 0.14	4.21 ± 0.34	101.0
	As	0.290 ± 0.010	0.285 ± 0.014	102.0
	Cd	0.0220 ± 0.0005	0.0224 ± 0.0013	98.2
	Co	0.0179 ± 0.0007	0.0177 ± 0.0005	101.1
	Cu	2.37 ± 0.10	2.35 ± 0.16	100.9
	Fe	7.38 ± 0.12	7.42 ± 0.44	99.5
	Hg	0.00582 ± 0.00027	0.00591 ± 0.00036	98.5
	K	1260 ± 10	1282 ± 11	98.3
	Mg	544 ± 7	559 ± 10	97.3
	Mn	19.5 ± 0.4	19.2 ± 1.8	101.6
	Na	6.63 ± 0.10	6.74 ± 0.19	98.4
	P	1550 ± 15	1530 ± 40	101.3
	Pb	0.0081 ± 0.0020	0.0080 ± 0.0030	101.3
	Se	0.355 ± 0.010	0.365 ± 0.029	97.3
	Sn	0.0051 ± 0.001	0.0050 ± 0.0010	102.0
	Zn	19.40 ± 0.12	19.42 ± 0.26	99.9
Lichen (IAEA-336)	As	0.64±0.04	0.63 ± 0.08	101.6
	Ba	6.2±0.9	6.4 ± 1.1	96.9
	Ce	1.30±0.07	1.28 ± 0.17	101.6
	Co	0.31±0.05	0.29 ± 0.05	106.9
	Cs	0.107±0.005	0.110 ± 0.013	97.2
	Cu	3.42±0.07	3.60 ± 0.50	95.0
	Fe	444±25	430 ± 50	103.3
	Hg	0.19±0.02	0.20 ± 0.04	95.0
	K	1790±120	1840 ± 200	97.3
	Mn	61.2±3.0	63.0 ± 7.0	97.1
	Na	335±150	320 ± 40	104.7
	Se	0.23±0.04	0.22 ± 0.04	104.5
	Sr	8.8±0.8	9.3 ± 1.1	94.6
	Zn	31.0±1.0	30.4 ± 3.4	102.0

[a] Mean (mean value of five measurements) ± standard deviation. [b] Confidence interval 95%.

Table S2: RDA, AI*, PTWI and PTMI* reference values for individual life stage groups defined by the IOM and the JECFA.

	**Essential minerals and trace elements**											
**Life stage group**	**Ca mg/day**	**Cr* μg/day**	**Cu μg/day**	**Fe mg/day**	**Mg mg/day**	**Mn* mg/day**	**Mo μg/day**	**P mg/day**	**Se μg/day**	**Zn mg/day**	**K* mg/day**	**Na* mg/day**
Men aged 19–30	1000	35*	900	8	400	2.3*	45	700	55	11	4700*	1500*
Women aged 19–30	1000	25*	900	18	310	1.8*	45	700	55	8	4700*	1500*
Pregnant women aged 19–30	1000	30*	1000	27	350	2.0*	50	700	60	11	4700*	1500*
Lactating women 19–30	1000	45*	1300	9	310	2.6*	50	700	70	12	5100*	1500*
Men aged 31–50	1000	35*	900	8	420	2.3*	45	700	55	11	4700*	1500*
Women aged 31–50	1000	25*	900	18	320	1.8*	45	700	55	8	4700*	1500*
Men aged 51–70	1000	30*	900	8	420	2.3*	45	700	55	11	4700	1300*
Women aged 51–70	1200	20*	900	8	320	1.8*	45	700	55	8	4700*	1300*
Men aged > 70	1200	30*	900	8	420	2.3*	45	700	55	11	4700*	1200*
Women aged > 70	1200	20*	900	8	320	1.8*	45	700	55	8	4700*	1200*

	**Toxic trace elements**			
**μg/kg of bw**	**Al**	**Sn**	**Hg**	**Cd***
PTWI	2000	14000	4	
PTMI*				25*

RDA – recommended dietary allowances, AI* – adequate intake (in case that the RDA value is not defined), IOM – Institute of Medicine, PTWI – provisional tolerable weekly intake, PTMI* – provisional tolerable monthly intake, bw – body weight, JFCEA – The Joint FAO/WHO Expert Committee on Food Additives.

Table S3: Dry matter and ash contents and dry matter digestibility (DMD) values of non-traditional wheat flakes.

**Wheat flakes**	**Dry matter (%)**	**Ash* (%)**	**DMD (%)**
Samtrot	92.4±0.6[a]	1.94±0.02[a]	89.2±0.7[a]
Tirol	93.2±0.4[b]	1.93±0.03[a]	87.4±0.4[b]
Dickkopf	93.8±0.4[c]	1.82±0.02[b]	90.5±0.5[c]
Rotkorn	91.8±0.5[d]	1.83±0.02[b]	88.8±0.3[d]
Megali	91.6±0.5[d]	1.89±0.03[c]	88.7±0.6[d]

All results are presented as means ± SD, n = 3 (the mean of three measurements). Means within a column with at least one identical small superscript do not differ significantly ($p \geq 0.05$).
*Ash content is presented on dry matter basis. DMD – dry matter digestibility value.

Table S4: Daily intakes for essential nutrients estimated from 100 g of non-traditional wheat flakes (women aged 19–30, pregnant and lactating women aged 19–30).

Analyt	Concentration range (mg/g)	Daily intake (mg/day)	% of RDA or AI* (women)	% of RDA or AI* (pregnant period)	% of RDA or AI* (lactating period)
Mg	0.658–0.967	65.8–96.7	21–31	19–28	21–31
P	0.129–0.172	12.9–17.2	2	2	2
K*	0.424–0.710	42.4–71.0	1–2*	1–2*	1*
Ca	0.141–0.244	14.1–24.4	1–2	1–2	1–2
Na*	0.00126–0.00551	0.13–0.55	< 0.1*	< 0.1*	< 0.1*
Mn*	0.0202–0.0329	2.02–3.29	112–183*	101–165*	78–127*
Fe	0.00746–0.0125	0.75–1.25	4–7	3–5	8–14
Cu	0.00379–0.00625	0.38–0.63	42–69	38–63	29–48
Zn	0.00607–0.00967	0.61–0.97	8–12	6–9	5–8
Cr*	0.0000605–0.0000745	0.0061–0.0075	24–30*	20–25*	13–17*
Mo	0.000282–0.000614	0.028–0.061	63–136	56–123	56–123
Se	0.0000208–0.0000563	0.0021–0.0056	4–10	3–9	3–8

RDA – recommended dietary allowances, AI* – adequate intake (in case that the RDA value is not defined).

Table S5: Daily intakes for essential nutrients estimated from 100 g of non-traditional wheat flakes (women aged 31–50, 51–70, and ≥ 70).

Analyt	Concentration range (mg/g)	Daily intake (mg/day)	% of RDA or AI* (women 31–50)	% of RDA or AI* (women aged 51–70)	% of RDA or AI* (women aged ≥ 70)
Mg	0.658–0.967	65.8–96.7	21–30	21–30	21–30
P	0.129–0.172	12.9–17.2	2	2	2
K*	0.424–0.710	42.4–71.0	1–2*	1–2*	1–2*
Ca	0.141–0.244	14.1–24.4	1–2	1–2	1–2
Na*	0.00126–0.00551	0.13–0.55	< 0.1*	< 0.1*	< 0.1*
Mn*	0.0202–0.0329	2.02–3.29	112–183*	112–183*	112–183*
Fe	0.00746–0.0125	0.75–1.25	4–7	9–16	9–16
Cu	0.00379–0.00625	0.38–0.63	42–69	42–69	42–69
Zn	0.00607–0.00967	0.61–0.97	8–12	8–12	8–12
Cr*	0.0000605–0.0000745	0.0061–0.0075	24–30*	30–37*	30–37*
Mo	0.000282–0.000614	0.028–0.061	63–136	63–136	63–136
Se	0.0000208–0.0000563	0.0021–0.0056	4–10	4–10	4–10

RDA – recommended dietary allowances, AI* – adequate intake (in case that the RDA value is not defined).

Table S6: Daily intake for essential nutrients estimated from 100 g of non-traditional wheat flakes (men aged 19–30, 31–50, 51–70, and ≥ 70).

Analyt	Concentration range (mg/g)	Daily intake (mg/day)	% of RDA or AI* (men 19–30)	% of RDA or AI* (men 31–50)	% of RDA or AI* (men 51–70)	% of RDA or AI* (men aged ≥ 70)
Mg	0.658–0.967	65.8–96.7	16–24	16–23	16–23	16–23
P	0.129–0.172	12.9–17.2	2	2	2	2
K*	0.424–0.710	42.4–71.0	1–2*	1–2*	1–2*	1–2*
Ca	0.141–0.244	14.1–24.4	1–2	1–2	1–2	1–2
Na*	0.00126–0.00551	0.13–0.55	< 0.1*	< 0.1*	< 0.1*	< 0.1*
Mn*	0.0202–0.0329	2.02–3.29	88–143*	88–143*	88–143*	88–143*
Fe	0.00746–0.0125	0.75–1.25	9–16	9–16	9–16	9–16
Cu	0.00379–0.00625	0.38–0.63	42–69	42–69	42–69	42–69
Zn	0.00607–0.00967	0.61–0.97	6–9	6–9	6–9	6–9
Cr*	0.0000605–0.0000745	0.0061–0.0075	17–21*	17–21*	20–25*	20–25*
Mo	0.000282–0.000614	0.028–0.061	63–136	63–136	63–136	63–136
Se	0.0000208–0.0000563	0.0021–0.0056	4–10	4–10	4–10	4–10

RDA – recommended dietary allowances, AI* – adequate intake (in case that the RDA value is not defined).

Table S7: Maximum dietary intake estimations of non-traditional wheat flakes to RDA (AI*), PTWI (PTMI*) values for essential and toxic elements.

	**Contribution to RDA or AI* (%)**											
Life stage group	Ca	Cr*	Cu	Fe	Mg	Mn*	Mo	P	Se	Zn	K*	Na*
men aged 19–30	2	21*	69	16	24	143*	136	2	10	9	2*	< 0.1*
Women aged 19–30	2	30*	69	7	31	183*	136	2	10	12	2*	< 0.1*
Pregnant women aged 19–30	2	25*	63	5	28	165*	123	2	9	9	2*	< 0.1*
Lactating women 19–30	2	17*	48	14	31	127*	123	2	8	8	2*	< 0.1*
Men aged 31–50	2	21*	69	16	23	143*	136	2	10	9	2*	< 0.1*
Women aged 31–50	2	30*	69	7	30	183*	136	2	10	12	2*	< 0.1*
Men aged 51–70	2	25*	69	16	23	143*	136	2	10	9	2*	< 0.1*
Women aged 51–70	2	37*	69	16	30	183*	136	2	10	12	2*	< 0.1*
Men aged > 70	2	25*	69	16	23	143*	136	2	10	9	2*	< 0.1*
Women aged > 70	2	37*	69	16	30	183*	136	2	10	12	2*	< 0.1*

	**Contribution to PTWI or PTMI* (%)**			
	Al	Sn	Hg	Cd*
60 kg	1.2	≤ 0.04	4.3	9.0*
70 kg	1.0	≤ 0.03	3.7	7.7*
80 kg	0.9	≤ 0.03	3.2	6.8*
90 kg	0.8	≤ 0.03	2.9	6.0*

Daily intake estimations were calculated from the maximum values of the concentrations for appropriate elements measured in non-traditional wheat flakes. Weekly (monthly*) intake estimations were calculated from the maximum values of the concentrations for appropriate elements measured in non-traditional wheat flakes. Intake levels were calculated for men and women of the average weight of 60–90 kg. A daily serving size of non-traditional wheat flakes was set to 100 g. RDA – recommended dietary allowances, AI* – adequate intake (in case that the RDA value is not defined). PTWI – provisional tolerable weekly intake, PTMI* – provisional tolerable monthly intake.

Table S8: Dietary intake estimations for toxic elements (based on bw value).

Analyt	Concentration range (μg/g)	Daily intake (μg)	Weekly/Monthly* intake (μg)	% of TPWI/PTMI* Bw (60 kg)	% of TPWI/PTMI* Bw (70 kg)	% of TPWI/PTMI* Bw (80 kg)	Bw (90 kg)
Al	1.45–2.01	145–201	1015–1407	0.8–1.2	0.7–1.0	0.6–0.9	0.6–0.8
Sn	0.107–0.467	10.7–46.7	74.9–237	≤ 0.04	≤ 0.03	≤ 0.03	≤ 0.03
Hg	0.0113–0.0148	0.13–1.48	7.91–10.4	3.3–4.3	2.8–3.7	2.5–3.2	2.2–2.9
Cd*	0.0056–0.0452	0.56–4.52	16.8–136	1.1–9.0	1.0–7.7	0.8–6.8	0.7–6.0

bw – body weight, PTWI – provisional tolerable weekly intake, PTMI* – provisional tolerable monthly intake.

Table S9: Daily intakes of non-essential and toxic elements.

Analyt	Concentration range (μg/g)	Concentration range (μg/g)
S	4.45–6.55	445–655
Ti	1.18–2.04	118–204
	**Concentration range (ng/g)**	**Daily intake (μg/day)**
Pb	34.4–57.8	3.44–5.78
Ni	62.8–208	6.28–20.8
Ba	218–1050	21.8–105
Sr	756–1430	75.6–143
V	17.4–112	1.74–11.2
Li	14.7–43.6	1.47–4.36
B	217–341	21.7–34.1
Sc	51.9–67.8	5.19–6.78
Zr	6.13–33.1	0.61–3.31
	**Concentration range (ng/g)**	**Daily intake (ng/day)**
As	5.86–10.9	586–1090
Ag	0.89–5.07	89–507
Co	6.11–15.5	611–1550
Sb	3.21–5.45	321–545
Tl	0.04–0.06	4–6
Be	0.04–0.11	4–11
Ga	9.25–15.0	925–1500
Ge	3.87–5.03	387–503
Y	0.35–0.72	35–72
Cs	0.28–1.35	28–135
Ce	0.60–6.88	60–688
Tb	0.02–0.21	2–21
Ho	0.02–0.09	2–9
Ta	6.10–9.83	610–983
Bi	0.73–1.76	73–176
U	0.13–0.58	13–58

A daily serving size of non-traditional wheat flakes was set to 100 g.

Table S10: Metal pollution index (MPI) for non-traditional wheat grains, flakes and undigested parts of flakes and the remaining parts of the metal pollution index (RP_{MPI}).

Samples	Native grains	Native flakes	Undigested part of flakes	Undigested part of flakes
	**MPI**			**RP_{MPI} (%)**
Samtrot	0.027 (100%)	0.018 (-33%)	0.035	21
Tirol	0.033 (100%)	0.023 (-30%)	0.042	23
Dickkopf	0.029 (100%)	0.021 (-28%)	0.033	15
Rotkorn	0.032 (100%)	0.024 (-25%)	0.044	21
Megali	0.024 (100%)	0.016 (-33%)	0.029	20

RP_{MPI} – remaining part of the metal pollution index which remains in undigested portion of flake.
